# Multi-objective energy management in a renewable and EV-integrated microgrid using an iterative map-based self-adaptive crystal structure algorithm

**DOI:** 10.1038/s41598-024-66644-3

**Published:** 2024-07-08

**Authors:** Arul Rajagopalan, Karthik Nagarajan, Mohit Bajaj, Sowmmiya Uthayakumar, Lukas Prokop, Vojtech Blazek

**Affiliations:** 1 Centre for Smart Grid Technologies, School of Electrical Engineering, Vellore Institute of Technology, Chennai, Tamilnadu 600 127 India; 2 Department of Electrical and Electronics Engineering, Hindustan Institute of Technology & Science, Chennai, Tamilnadu 603 103 India; 3 Electrical Engineering Department, Graphic Era (Deemed to Be University), Dehradun, 248002 India; 4 Hourani Center for Applied Scientific Research, Al-Ahliyya Amman University, Amman, Jordan; 5 Graphic Era Hill University, Dehradun, 248002 India; 6 Department of Electrical Engineering, SRM Institute of Science and Technology, Kattankulathur, Tamilnadu 603203 India; 7 ENET Centre, VSB—Technical University of Ostrava, 708 00 Ostrava, Czech Republic

**Keywords:** Energy management, Iterative map-based self-adaptive crystal structure algorithm, Electric vehicles, Renewable energy sources, Microgrid, Optimal scheduling, Wind power, Solar photovoltaic, Engineering, Electrical and electronic engineering

## Abstract

The use of plug-in hybrid electric vehicles (PHEVs) provides a way to address energy and environmental issues. Integrating a large number of PHEVs with advanced control and storage capabilities can enhance the flexibility of the distribution grid. This study proposes an innovative energy management strategy (EMS) using an Iterative map-based self-adaptive crystal structure algorithm (SaCryStAl) specifically designed for microgrids with renewable energy sources (RESs) and PHEVs. The goal is to optimize multi-objective scheduling for a microgrid with wind turbines, micro-turbines, fuel cells, solar photovoltaic systems, and batteries to balance power and store excess energy. The aim is to minimize microgrid operating costs while considering environmental impacts. The optimization problem is framed as a multi-objective problem with nonlinear constraints, using fuzzy logic to aid decision-making. In the first scenario, the microgrid is optimized with all RESs installed within predetermined boundaries, in addition to grid connection. In the second scenario, the microgrid operates with a wind turbine at rated power. The third case study involves integrating plug-in hybrid electric vehicles (PHEVs) into the microgrid in three charging modes: coordinated, smart, and uncoordinated, utilizing standard and rated RES power. The SaCryStAl algorithm showed superior performance in operation cost, emissions, and execution time compared to traditional CryStAl and other recent optimization methods. The proposed SaCryStAl algorithm achieved optimal solutions in the first scenario for cost and emissions at 177.29 €ct and 469.92 kg, respectively, within a reasonable time frame. In the second scenario, it yielded optimal cost and emissions values of 112.02 €ct and 196.15 kg, respectively. Lastly, in the third scenario, the SaCryStAl algorithm achieves optimal cost values of 319.9301 €ct, 160.9827 €ct and 128.2815 €ct for uncoordinated charging, coordinated charging and smart charging modes respectively. Optimization results reveal that the proposed SaCryStAl outperformed other evolutionary optimization algorithms, such as differential evolution, CryStAl, Grey Wolf Optimizer, particle swarm optimization, and genetic algorithm, as confirmed through test cases.

## Introduction

Microgrids have become a cutting-edge method for tackling the challenges of contemporary energy systems, providing targeted and flexible capabilities for generating, distributing, and managing energy^1,2^. Microgrids, in contrast to conventional centralized grids, are decentralized networks capable of functioning alone or in tandem with the primary grid, offering enhanced resilience, reliability, and efficiency^3,4^. The incorporation of renewable energy sources (RESs), such as solar photovoltaics (PV) and wind turbines (WT), has played a crucial role in the advancement of microgrids^5,6^. Renewable energy sources provide environmentally friendly and sustainable methods of generating energy, hence decreasing dependence on fossil fuels and minimizing the release of greenhouse gases^7^. Furthermore, advancements in energy storage technologies, such as lithium-ion batteries and pumped hydro storage, have significantly enhanced the capacity of microgrids to store excess energy for subsequent use^8,9^. This advancement has led to a more stable power grid and improved integration of intermittent renewable sources^10,11^. The emergence of microgrid technology has paralleled the growing adoption of Plug-In Hybrid Electric Vehicles (PHEVs), presenting both opportunities and challenges in energy management^12,13^. PHEVs serve as both efficient and environmentally friendly modes of transportation, while also serving as mobile energy storage units^14,15^. When incorporated into microgrid operations, plug-in hybrid electric vehicles can actively engage in demand response programs, offer assistance to the grid, and function as alternative power sources in times of emergencies^16,17^. Addressing multi-objective energy management within a microgrid incorporating plug-in electric vehicles (PEVs) represents a crucial and intricate challenge within the realm of energy systems^18,19^. A microgrid is defined as a localized aggregation of electrical loads and distributed energy resources capable of functioning either in a grid-connected or standalone capacity^20–22^. PEVs are becoming increasingly popular as a means of reducing carbon emissions and dependency on fossil fuels^23^. The integration of PEVs into a microgrid creates a new set of challenges and opportunities for energy management^24,25^. PEVs offer the advantage of serving as mobile energy storage units, contributing flexibility and resilience to the microgrid^26^. However, the charging and discharging of PEVs require careful management to fulfill the energy demands of the microgrid while also addressing the requirements of individual PEV owners^27,28^. Multi-objective energy management in a microgrid incorporating PEVs entails the optimization of multiple competing objectives, including minimizing energy expenses, mitigating greenhouse gas emissions, and guaranteeing a dependable and resilient power provision^29–31^. This problem requires sophisticated algorithms and models that can handle the complexity and uncertainty of energy systems. Overall, multi-objective energy management in a microgrid with the integration of PEVs is an important and challenging problem that requires interdisciplinary research and collaboration between experts in energy systems, optimization, and control theory^32–36^. Its successful implementation can lead to significant benefits, including reduced energy costs, increased energy efficiency, and reduced carbon emissions^37,38^.

In^39^, a multi-objective economic dispatch model for microgrids incorporating electric vehicles and transferable loads was implemented. Simulation was carried out on four different case studies. The objective functions under consideration included the operational cost of the microgrid, the utilization rate of photovoltaic energy, and the power fluctuation between the microgrid and the utility^40^. A two-stage optimization strategy was implemented to perform the environmental and economic scheduling of microgrid with the integration of electric vehicles^41^. In our previous study^42^, we conducted multi-objective energy management in a microgrid integrating plug-in electric vehicles. The model suggested provided a state of charge curve for microgrids, considering the state of charge limits of plug-in electric vehicle batteries to prevent overcharging and over-discharging. Additionally, an enhanced grey wolf algorithm was proposed to address the multi-objective energy management problem. Moreover, in^43^, an adaptive simulated annealing particle swarm optimization algorithm (ASAPSO) was introduced for the multi-objective optimal scheduling of microgrids incorporating electric vehicles. The objective functions considered were operational cost and emissions. To strike a better balance between these objectives, coordination of renewable energy consumption and load management was achieved using the linear weighting method, grounded on a two-player zero-sum game. Microgrid energy management strategies with peak load reduction (PLR)-based demand response program was proposed to lower end-user energy costs and lower the peak load demand on the power grid^44^. The optimal management of a microgrid equipped with renewable energy sources and electric vehicles (EVs) alongside responsive loads has been undertaken to achieve cost savings and emissions reduction^45^. To address uncertainties stemming from wind turbine (WT) and photovoltaic (PV) power generation, a demand response program (DRP) was devised to manage required grid reserves. Furthermore, in^46^, an optimal microgrid operation considering charging patterns for plug-in hybrid electric vehicles (PHEVs) was proposed. To regulate the charging and discharging processes of PHEVs within the microgrid, along with responsive loads, a smart charging approach was recommended^46^. The study in^47^ delved into the stochastic operation planning of a microgrid (MG) incorporating Battery Energy Storage System (BESS), renewable energies, and non-renewable energy sources. They devised a stochastic optimization model with a sole objective and proposed employing a hybrid approach combining the whale optimization algorithm with the pattern search algorithm to tackle the optimization challenge. An ideal energy management system for microgrids, incorporating distributed generation and electric vehicles, was proposed in^48^, aiming to reduce operational expenses and environmental pollutants. The optimization approach accounts for the performance of electric vehicles in both petrol and electric modes. In another study^49^, a scenario-based stochastic management approach is utilized to achieve the optimal operation of a multi-carrier microgrid (MG). This microgrid incorporates various components such as a wind turbine, photovoltaic panel, fuel cell, microturbine, boiler, combined heat and power unit, along with electrical, thermal, and hydrogen loads, as well as storage facilities for electrical energy, hydrogen, and thermal energy. To further reduce overall running costs, a novel approach for scheduling electric vehicles and battery storage in tandem while considering the demand response program (DRP) is proposed in^50^. Additionally, the impact of DRP collaboration and optimal scheduling of electric vehicles and energy storage systems on operational expenses, power transactions with the upstream grid, hourly distributed energy resources, and system technical parameters is explored. Finally, in^51^, a two-stage energy management framework employing stochastic chance constraint model predictive control (MPC) is introduced to solve the microgrid energy management problem with the integration of electric vehicles. The framework adopts a mixed-stage optimization approach, gradually optimizing the problem across various time scales. A detailed investigation into energy management systems (EMS) for microgrids was carried out, emphasizing EMS components and the optimization methodologies integrated into the EMS framework. Extensive literature review on microgrid energy management systems (EMS) was performed, categorizing them according to four criteria: the optimization methods employed, the grid type under consideration, the microgrid’s operational mode (connected to the main grid or operating independently), and the software/solvers used as a basis for addressing EMS challenges^52^. An oppositional gradient-based grey wolf optimizer (OGGWO) was proposed to implement the multi-objective optimal scheduling of a microgrid^53^. Table 1 presents an overview of the research contributions in microgrid energy management covering objective functions, optimization methods, test system components, and notable remarks. Since its inception, the crystal structure algorithm has gained widespread popularity due to its remarkable adaptability, simple structure, and lack of predefined parameters. Despite CryStAl’s superior performance in several areas, the crystal structure algorithm still has certain flaws. There is not enough exploration since CryStAl is sensitive to local extremes during iteration. To address the limitations of the crystal structure algorithm, we propose the Iterative Map Self-Adaptive Crystal Structure Algorithm (SaCryStAl). The efficacy of the proposed optimization technique was examined across three distinct scenarios to assess its performance.

**Table 1:** Exploring optimization strategies for energy management in microgrid: a review.

References	Year	Components of test system used	Objective functions	Methodology	Remarks
^54^	2024	WT, PV, battery, MT, diesel generator, FC	Operation cost	Intelligent golden jackal optimization	Integration of electric vehicle is not considered
^55^	2023	PV, battery, MT, thermal generator, CHP	Operation cost, emission	Epsilon constraint algorithm	Integration of WT and FC not considered
^56^	2024	WT, PV, battery, MT, diesel generator, FC	Operation cost, emission	Manta ray foraging optimization	Analysis of environmental pollution is ignored. Multi-objective optimization not implemented
^57^	2023	CES, EES, CAES, EHP, AC, heat pump	Operation cost, emission	Blue whale optimization algorithm	Integration of WT, PV, and MT not considered. Different charging modes of EV not analyzed
^58^	2023	PV, WT, battery	MG and EV cost	Enhanced variant multi-objective particle swarm optimization algorithm	Analysis of environmental pollution is ignored
^59^	2023	CHP, gas boiler, WT, PV, HS, BS	Operating cost of multi-microgrid, profit of the distribution company	Mixed-integer linear programming, ε-constraint approach, mixed-integer nonlinear programming	Analysis of environmental pollution is ignored
^60^	2023	WT, PV, battery, MT, diesel generator, FC and grid	Operation cost, emission	Improved shuffled frog leaping algorithm	Different charging modes of EV not analyzed
^61^	2023	thermal generators, battery and grid	operation cost, emission	efficient black widow optimization algorithm,	Integration of renewable energy sources is ignored
^62^	2023	PV, diesel generator, grid and battery	Energy consumption, life cycle of battery, practicality of the renewable energy usage	Extended optimal ε-variable technique	Analysis of operating cost and emission is ignored
^63^	2024	Battery, supercapacitor	Battery capacity loss, state of charge	NSGA-III,	Integration of renewable energy sources is ignored
^64^	2023	WT, MT, PV, FC and battery	Generation cost, penalty cost of frequency overrun	Back Propagation neural network	Analysis of environmental pollution is ignored
^65^	2023	PV, WT, CHP, boiler, battery	Operation cost, emission	Lexicography-compromised programming	Integration of MT and FC is ignored
^66^	2022	WT, PV	Voltage deviation, energy not supplied, overall annual cost of energy in a microgrid	Jellyfish search optimizer	Integration of MT and FC is ignored
^67^	2024	PV, battery	Electricity consumption costs, variability in grid-side energy supply	Multi-objective particle swarm algorithm	Analysis of environmental pollution is ignored
^68^	2024	WT, PV, diesel generator, MGT, battery	Operation cost, emission	Improved PSO algorithm	Integration with EV is ignored
^69^	2023	PV, WT, battery	Operating cost, voltage deviation, active power loss	Wavelet neural network	Analysis of environmental pollution is ignored
^70^	2023	WT, PV	Operating cost, rate of renewable energy, cost of the distribution network operators, cost of electric vehicle users, profit of microgrid operators	Improved PSO algorithm	Analysis of environmental pollution is ignored. Integration of MT and FC is ignored
^71^	2024	WT, PV, battery and grid	Operation cost, emission, voltage deviation, active power loss	Multi-objective artificial vultures optimization algorithm	Integration of MT and FC is ignored
^72^	2023	WT, PV, battery	Operating cost	PSO	Analysis of environmental pollution is ignored. Integration of MT and FC is ignored
^73^	2023	WT, PV, battery	Cost of electric vehicle aggregator	Twin delayed deep deterministic policy gradient algorithm	Analysis of environmental pollution is ignored. Integration of MT and FC is ignored

The contribution to the knowledge section of this paper lies in several key areas. Firstly, we introduce a novel energy management technique tailored specifically for microgrids (MGs) integrated with renewable energy sources (RESs) and Plug-In Hybrid Electric Vehicles (PHEVs). This technique utilizes the SaCryStAl algorithm, which efficiently distributes energy among various units within the grid-connected MG. Secondly we address the dual objectives of minimizing MG operating costs and reducing pollutant emissions, a critical consideration in contemporary energy systems. By formulating an objective function that accounts for both economic and environmental factors, we provide a comprehensive framework for optimizing MG operation. Thirdly, we compare the performance of our proposed algorithm with existing evolutionary optimization approaches, demonstrating its superiority in terms of stability, convergence, and performance. Additionally, we present a true collection of Pareto-optimal solutions, offering system operators a range of options to tailor power dispatch strategies according to their economic and environmental objectives. Lastly, our study highlights the impact of widespread PHEV and RES adoption on grid functioning, underscoring the need for advanced optimization techniques in managing these complex systems. Overall, our contributions advance the field of sustainable energy management by providing practical insights and effective solutions for optimizing MG operation in the context of evolving energy landscapes.

The primary contributions of this paper can be outlined as follows:

- Presenting a multi-objective framework for the short-term scheduling of a microgrid (MG) incorporating a plug-in hybrid electric vehicle (PHEV), with cost and emissions as dual objective functions.
- Incorporating the proposed SaCryStAl optimization technique to simultaneously minimize costs and emissions, generate Pareto optimal solutions, and determine the optimal compromise solution using a fuzzy satisfaction method.
- The proposed SaCryStAl is investigated on three different scenarios including the operation of PHEVs’ in three different modes.
- The proposed SaCryStAl algorithm produced exceptional results when compared to recently published publications in terms of cost, emission, and solution time.

The rest of the paper is structured as follows:

In section “Iterative map-based self-adaptive crystal structure algorithm (SaCryStAl)”, we delve into the implementation of the proposed Iterative map-based Self-Adaptive Crystal Structure Algorithm (SaCryStAl) to address the multi-objective energy management problem. Section “Modeling of a microgrid test system” is dedicated to the modeling of the microgrid test system under consideration. Section “Problem formulation” outlines the formulation of the multi-objective energy management problem aimed at minimizing operating costs and emissions. In section “Fuzzy logic-based selection of optimal compromise solution”, we elaborate on the formulation of fuzzy logic assortment for determining the optimal compromise solution. The concept of microgrid modelling is covered in section “Uncertainty models for wind and solar energy”. Lastly, section “Modeling of microgrid” presents a comprehensive demonstration of the superior performance and feasibility of the proposed SaCryStAl algorithm, juxtaposed with other meta-heuristic optimization algorithms such as FSAPSO, KH, PSO, WOA, GA and GWO.

## Iterative map-based self-adaptive crystal structure algorithm (SaCryStAl)

### Classical crystal structure algorithm (CryStAl)

The mathematical model of CryStAl, which applies the fundamental ideas of crystal formulation with the appropriate adjustments, is described in this part. All possible solutions to the optimization procedure are viewed as individual crystals in the solution space in this model. Initial crystals are generated randomly^74^. The idea of adding a basis to lattice points to create crystals served as the inspiration for the crystal structure algorithm. Siamak Talatahar suggested this crystal structure algorithm in 2021 based on this idea^75^.

The initial population is randomly generated within the bounds using Eq. (1).

1 $$X_{i,j} = L_{i,j} + r*\left( {U_{i,j} - L_{i,j} } \right), \,\,\,\,\,{\text{i}} = {1},{2},...,\,\,\,\,\,N, {\text{j}} = {1},{2},...,{\text{m}}.$$

where *X*_i,j_ is the jth variable in the ith solution vector, where m is the problem’s dimension and *N* is the number of crystals or potential solutions. “$r$ “ is a random number between [0, 1], *L*_*j*_ and *U*_*j*_ are the variables, lower and upper bounds. The structure of the initial population matrix is shown in Eq. (2).

2 $${\text{C}} = \left[ {\begin{array}{*{20}c} {C_{i1} } \\ {C_{i2} } \\ \vdots \\ {C_{iN} } \\ \end{array} } \right] = \left[ {\begin{array}{*{20}c} {x_{1,1} } & \cdots & {x_{1,m} } \\ {x_{2,1} } & \ldots & {x_{2,m} } \\ \vdots & \cdots & \vdots \\ {x_{N,1} } & \ldots & {x_{N,m} } \\ \end{array} } \right],\,\,\,\,\,\,\,{\text{i}} = {1},\,{2}, \ldots ,\,N\& {\text{ j}} = {1},\,{2}, \ldots ,{\text{m}}.$$

Based on the concept of ‘basis’ in crystallography, all the crystals at the corners are considered as the main crystals (${\text{C}}_{rM}$). The crystal with the best fitness value is taken as ${\text{ C}}_{rb}$ and the mean values of randomly-selected crystals are denoted by $F_{c}$. The new crystals are generated in the search space by using the following Eqs. (3–6). This process will be repeated for N number of iterations considered.

(i) Simple cubicle:
  3 $${\text{C}}_{rN} = {\text{C}}_{rO} + r {\text{C}}_{rM}$$
(ii) Cubicle with the best crystals:
  4 $${\text{C}}_{rN} = {\text{C}}_{rO} + r_{1} {\text{C}}_{rM} + \,r_{2} {\text{C}}_{rb} ,\,\,\,{\text{where}}\,\,r_{1} = \left( {1 - r} \right) \& \,r_{2} = r_{1} /{\text{N}}$$
(iii) Cubicle with the mean crystals:
  5 $${\text{C}}_{rN} = {\text{C}}_{rO} + r_{1} {\text{C}}_{rM} + r_{2} F_{c}$$
(iv) Cubicle with the best and mean crystals:
  6 $${\text{C}}_{rN} = {\text{C}}_{rO} + r_{1} {\text{C}}_{rM} + \,r_{2} {\text{C}}_{rb} + r_{3} F_{c} ,\,\,\,\,{\text{where}}\,\,r_{3} = r_{2} /{\text{N}}$$

where, in the four equations above, ${\text{C}}_{rN}$ is the new position, ${\text{C}}_{rO}$ is the old position, and *r*, $r_{1}$, $r_{2}$ and $r_{3}$ are random numbers related to one another.

### Steps involved in the proposed Iterative map-based self-adaptive crystal structure algorithm

*Step 1* Generate N number of initial crystals $\left( {X_{i,j} } \right)$ using the Eq. (1) and find the opposite values for all the crystals $\left( {\overline{X}_{ij} } \right)$ using the Eq. (7).

7 $$\overline{X}_{ij} = U_{{\text{i}}} + L_{{\text{i}}} - X_{ij} ,\,\,\,\,\,\,\,\,\,{\text{i}} = {1},{2}, \ldots ,{\text{N}} \& {\text{j}} = {1},{2}, \ldots ,{\text{n}}$$

Calculate the fitness function value for all the crystals, arrange them in ascending order, and take the first N as the initial population size.

*Step 2* Generate the random (*r*) value using an iterative map ^76^ Eq. (8).

8 $$r_{n + 1} = {\text{sin }}\left( {{\text{a }}/ r_{n} } \right){ }$$

‘*a*’ represents a parameter that can be adjusted. Its value ranges from 0 to α. Based on our experience, the optimal value of ‘*a*’ is fixed as 0.48 and the starting rand ($r_{n}$) value is 0.26.

**Figure 1:**
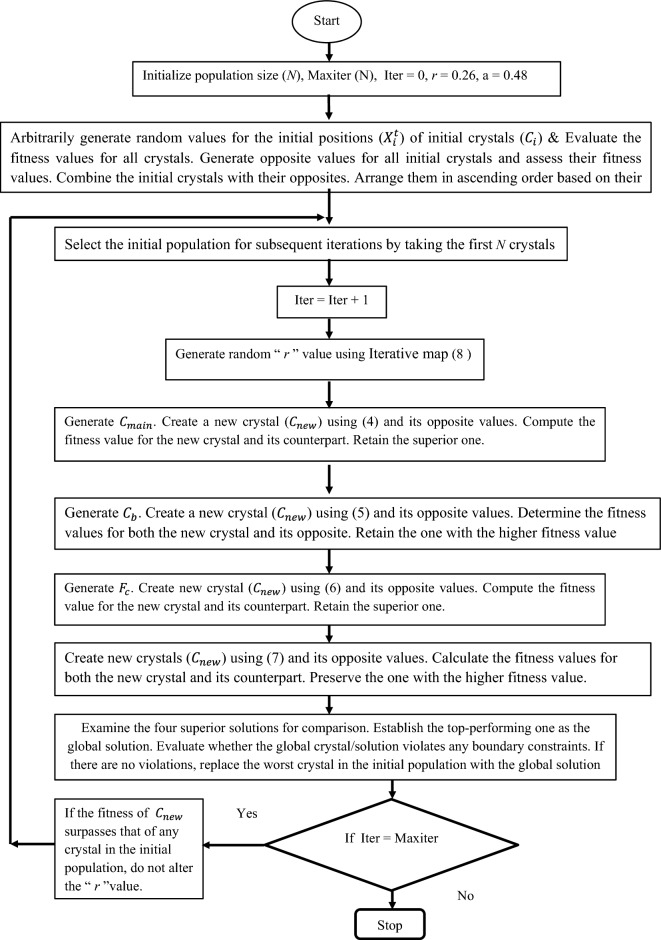
Flowchart outlining the SaCryStAl algorithm.

*Step 3* Generate four new crystals using Eqs. (3–6) and find their opposite values using Eq. (7). Select the best crystal out of the eight newly created values and compare its fitness value with the fitness values of the initial population. If the best crystal replaces any one of the worst crystals in the initial population then maintain the $r_{n}$ values in the Eq. (3). Otherwise, generate a new value $r_{n}$ using Eq. (8). The detailed working mechanism of the proposed algorithm is given in Fig. 1.

### *Pseudo*-code of SaCryStAl

Initializing the positions of crystals $C_{ij}$ and $\overline{C}_{ij}$ using (1) & (7).

Calculate the fitness value for all the crystals.

Generate random “ *r* “ value using Eq. (8)

while N < N_max_ do.

for (every crystal) do.

Create $${\text{C}}_{rM}$$.

Create new crystals through formula (3) and its opposite value.

Generate C_rb_.

Construct new crystals through formula (4) and its opposite value.

Create $$F_{c}$$.

Create new crystals through formula (5) and its opposite value.

Create new crystals through formula (6) and its opposite value.

if (all the newly created crystal exceeds the limits) then adjust the location of the new crystal.

end if.

Compute the fitness values for all the newly generated crystals.

Revise the population with the best fitness value.

end for.

N = N + 1.

end while.

Display the best crystal.

To assess the efficacy of the suggested algorithm, we select five standard mathematical test functions and proceed with its implementation. The outcomes from our method surpass those of the conventional approach and other methods referenced in Ref.^77^, while matching the performance of the ABC method in terms of quality. Table 2 presents the test data from applying the classical and proposed algorithm to benchmark functions.

**Table 2:** Exploration of the proposed SaCryStAl algorithm to benchmark test functions.

S.No	Name of the function	Objective function	Characteristics	Dime-nsions	Range	Method	f_op_	Mean	Std.Dev
1	Sphere	*f*_1_(*x*) = $\mathop \sum \limits_{i = 1}^{n} x_{i}^{2}$	Unimodal separable	30	[− 100,100]	CM	0.00982	0.0693	0.2736
						PM	0	0	0
2	Schwefel 1.2	*f*_2_(*x*) = $\mathop \sum \limits_{i = 1}^{n} \left( {\mathop \sum \limits_{j = 1}^{i} x_{j} } \right)^{2}$	Unimodal non-separable	30	[− 100,100]	CM	0.00875	0.0946	0.0438
						PM	0	0	0
3	Rosenbrock	*f*_3_(*x*) = $\mathop \sum \limits_{i = 1}^{n - 1} \left( {100\left( {x_{i + 1} - x_{i}^{2} } \right)^{2} } \right) + \left( {x_{i} - 1} \right)^{2}$	Unimodal non-separable	30	[− 30,30]	CM	0.00948	1.096897	0.09576
						PM	0	0.0887707	0.077390
4	Quartic	*f*_4_(*x*) = $\mathop \sum \limits_{i = 1}^{n} ix_{i}^{4} + {\text{random}}\left[ {0,{1}} \right)$	Unimodal separable	30	[− 1.28,1.28]	CM	0.00264	0.04858	0.02623
						PM	0	0.030017	0.004868
5	Rastrigin	*f*_*5*_*(x)* = $\mathop \sum \limits_{{{\text{i}} = 1}}^{{\text{n}}} \left[ {{\text{x}}_{{\text{i}}}^{2} - {1}0\,{\text{cos}}\,\left( {{2}\pi {\text{x}}_{{\text{i}}} } \right) + {1}0} \right]$	Multimodal separable	30	[− 5.12,5.12]	CM	0.00035	0.000257	0.000537
						PM	0	0	0

*CM* classical method & *PM* proposed method.

## Modeling of a microgrid test system

This research investigates a grid-connected microgrid (MG) comprising a wind turbine (WT), photovoltaic (PV) array, microturbine (MT), fuel cell (FC), storage battery, plug-in hybrid electric vehicles (PHEVs), and loads, connected to the main grid via a 20 kV/400 V transformer^78,79^. The microgrid configuration under study is adapted from the topology outlined in Ref.^78^. The PHEV represents a unique vehicle with various charging options and a transportation system enabling the use of fossil fuels during extended journeys if the battery’s charge depletes^80,81^. Factors such as the state of charge (SOC) of the PHEV’s battery, charger size, charging duration, and vehicle volume influence its charging rate^82,83^. Considering the unpredictable charging demands of these vehicles, our research encompasses multiple charging methods—coordinated, uncoordinated, and smart charging—to comprehensively characterize this phenomenon^84,85^.

In the initial charging pattern being examined, termed uncoordinated charging, the plug-in hybrid electric vehicles (PHEVs) have the flexibility to connect to the grid for charging at any time they desire^86,87^. These vehicles typically undertake two daily trips, with the first occurring in the morning and the second in the evening as they return home. Upon arriving home at 6:00 PM, it is assumed that the vehicles have the opportunity to connect to the grid for charging purposes. The probability density function (PDF) can be used to develop this^88^ as follows:

9 $$f\left( {t_{start} } \right) = \frac{1}{b - a} a \le t_{start} \le b a = 18,\, b = 19$$

where *a* and *b* represent constants referring to the time.

In the coordinated charging pattern, owners of plug-in hybrid electric vehicles (PHEVs) opt to connect their vehicles to the grid during off-peak hours to circumvent peak times and associated high prices. Consequently, they typically initiate charging sessions after 9:00 PM. This preference for off-peak charging is articulated in^88^.

10 $$f\left( {t_{start} } \right) = \frac{1}{b - a} a \le t_{start} \le b a = 21, \,b = 24$$

In a smart charging pattern, PHEVs are connected to the grid when power prices are at their lowest or when energy is abundant^89^. This pattern can be represented using a standard PDF as seen below^88^:

11 $$f\left( {t_{start} } \right) = \left( {\frac{1}{{\sigma \sqrt {2\pi } }}} \right)e^{{0.5\left( {\frac{{t_{start} - \mu }}{\sigma }} \right)^{2} }} \mu = 1, \,\sigma = 3$$

where $\mu$ and $\sigma$ represent the standard deviation and mean respectively.

With the use of all-electric range (AER), the SOC of PHEVs during the charging process may be calculated as follows:

12 $$SOC = \left\{ {\begin{array}{*{20}l} {0 m > AER} \hfill \\ {\frac{AER - m}{{AER}} \times 100m \le AER} \hfill \\ \end{array} } \right.$$

Here, m represents the mileage of the PHEV in miles. Our study focuses on the PHEV-20 model, and charger availability data is sourced from^90^. We illustrate the charging process in residential areas using level 1 and level 2 chargers, which are the focus of this research. Level 3 chargers are designated for commercial and public transportation purposes.

## Problem formulation

In this study, we introduce a precise mathematical model for short-term energy management aimed at minimizing operating costs and pollution emissions within a microgrid. Achieving optimal performance in a microgrid involves utilizing a multi-objective optimization approach. The key aim of multi-objective energy management in a typical microgrid setting is to identify the best power generation levels and determine the suitable operational states (ON or OFF) for distributed generation units. This process must optimize both the microgrid’s operating costs and its net emissions, all while complying with predefined equality and inequality constraints. This study introduces a detailed mathematical model tailored for short-term energy management, aiming to cut costs and reduce emissions within the microgrid.

### Objective functions

Optimizing both cost and emissions in a grid-connected microgrid is essential for balancing economic efficiency, environmental sustainability, regulatory compliance, and social responsibility. By targeting these goals simultaneously, microgrid operators can enhance their operations to benefit stakeholders and society at large. This study examines two key objective functions: operational costs and pollution emissions.

#### Operating cost

Operational costs form a foundational aspect of energy management strategies, significantly influencing their effectiveness and efficiency. These costs play a vital role in ensuring the economic sustainability and viability of microgrid operations^91^. Total operational expenses for the microgrid, calculated in Euro cents (€ct), encompass fuel costs for distributed generation units, startup and shutdown expenses, and costs associated with power exchange between the utility and the microgrid. The aim of managing overall operating costs is to achieve optimal power flow from energy sources to load centers over a given period, while prioritizing cost-effectiveness.

Operational costs contribute to bolstering the resilience and stability of microgrid systems. By accounting for factors such as fuel and maintenance expenses and penalties for deviations from operational constraints, these costs help identify robust energy management strategies that can endure uncertainties and disturbances, ensuring a reliable and continuous power supply^91^.

13 $$\min f_{T.C} \left( X \right) = \mathop \sum \limits_{h}^{T} \left( {TC_{DG}^{h} + TC_{ST}^{h} + TC_{GR}^{h} } \right)$$

14 $$TC_{DG}^{h} = C_{PV}^{h} + C_{WT}^{h} + C_{MT}^{h} + C_{FC}^{h}$$

15 $$C_{PV}^{h} = U_{PV} \left( h \right) \cdot P_{PV} \left( h \right) \cdot B_{PV} \left( h \right) + S_{PV} \cdot \left| {U_{PV} \left( h \right) - U_{PV} \left( {h - 1} \right)} \right|$$

16 $$C_{WT}^{h} = U_{WT} \left( h \right) \cdot P_{WT} \left( h \right) \cdot B_{WT} \left( h \right) + S_{WT} \cdot \left| {U_{WT} \left( h \right) - U_{WT} \left( {h - 1} \right)} \right|$$

17 $$C_{MT}^{h} = U_{MT} \left( h \right) \cdot P_{MT} \left( h \right) \cdot B_{MT} \left( h \right) + S_{MT} \cdot \left| {U_{MT} \left( h \right) - U_{MT} \left( {h - 1} \right)} \right|$$

18 $$C_{FC}^{h} = U_{FC} \left( h \right) \cdot P_{FC} \left( h \right) \cdot B_{FC} \left( h \right) + S_{FC} \cdot \left| {U_{WT} \left( h \right) - U_{FC} \left( {h - 1} \right)} \right|$$

19 $$CT_{ST}^{h} = U_{BT} \left( h \right) \cdot P_{BT} \left( h \right) \cdot B_{BT} \left( h \right) + S_{BT} \cdot \left| {U_{BT} \left( h \right) - U_{BT} \left( {h - 1} \right)} \right|$$

20 $$CT_{GR}^{h} = P_{GR} \left( h \right) \cdot B_{GR} \left( h \right)$$

At hour $h$, the variables $U_{PV}$, $U_{WT}$, $U_{MT}$, $U_{FC}$, and $U_{BT}$ represent the operating states of the solar photovoltaic system, wind turbine, microturbine, fuel cell, and battery, respectively. Similarly, the bids for distributed generators (DGs), storage devices, and the grid at hour hr are denoted by $B_{PV} ,\, B_{WT}$, $B_{MT}$, $B_{FC}$, $B_{BT}$ and $B_{GR}$. The power outputs of the solar photovoltaic, wind turbine, microturbine, fuel cell, and battery unit at time $h$ are represented by $P_{PV} \left( h \right)$, $P_{WT} \left( h \right), P_{MT} \left( h \right), P_{FC} \left( h \right)$ and $P_{BT} \left( h \right)$ respectively. The start-up and shut-down costs of the solar photovoltaic, wind turbine, microturbine, fuel cell, and battery units are indicated by $S_{PV} , S_{WT}$, $S_{MT}$, $S_{FC}$ and $S_{BT}$ respectively. Additionally, $P_{GR} \left( h \right)$ denotes the quantity of power traded with the market at hour hr, as referenced in^74,91^.

This study focuses on the design variables, which include the generated power outputs and the operating states of the generation units. The decision variables, consisting of the active power of the units and their corresponding states, are represented by the vector $X$, as defined in^91^.

21 $$X = \left[ {P_{DG} ,P_{ST} , U_{DG, } U_{ST} } \right]$$

22 $$P_{DG} = \left[ {P_{DG1} ,P_{DG2} , \ldots , P_{DGi} , P_{GRD} } \right] \forall \in N_{DG}$$

23 $$P_{ST} = \left[ {P_{ST1} ,P_{ST2} , \ldots , P_{STj} } \right] \forall \in N_{ST}$$

24 $$U_{DG} = \left[ {U_{DG1} ,U_{DG2} , \ldots , U_{DGi} } \right]$$

25 $$U_{ST} = \left[ {U_{ST1} ,U_{ST2} , \ldots , U_{STj} } \right]$$

Here $N_{DG}$ characterizes the number of distributed generators (DGs) installed in the microgrid (MG), whilst $N_{ST}$ signifies the number of storage units.

#### Objective function for pollution

The objective function for emissions is essential for evaluating the environmental impact of microgrid operations^92^. Microgrids emit pollutants due to various components such as the grid, generation units, and energy storage resources^93^. By quantifying emissions of pollutants such as CO_2_, SO_2_, and NO_x_, this function provides a comprehensive measure of the ecological footprint of energy generation and consumption within the microgrid. This is particularly significant in addressing climate change and mitigating air pollution, as it allows stakeholders to monitor and reduce the environmental effects of energy production^94^. The emissions objective function plays a crucial role in aligning energy management strategies with regulatory standards and sustainability goals. By incorporating emissions considerations into the optimization process, it supports compliance with emissions regulations and fosters proactive environmental stewardship. This helps microgrid operators avoid potential penalties and regulatory challenges while positioning them as leaders in promoting clean energy practices. Additionally, the emissions objective function enhances the overall efficiency and resilience of microgrid systems. By optimizing energy management strategies to minimize emissions while meeting operational needs, it encourages the adoption of cleaner and more sustainable technologies. The mathematical formula for calculating emissions, including nitrogen dioxide (NO_x_), carbon dioxide (CO_2_), and Sulfur dioxide (SO_2_), is presented below^91^.

Min

26 $$\min f_{T.E} \left( X \right) = \mathop \sum \limits_{h = 1}^{T} \left\{ {\mathop \sum \limits_{i = 1}^{{N_{DG} }} \left[ {U_{i} \left( h \right)P_{DGi} \left( h \right)E_{DGi} \left( h \right)} \right] + \mathop \sum \limits_{j = 1}^{{N_{ST} }} \left[ {U_{j} \left( h \right)P_{STj} \left( h \right)E_{STj} \left( h \right)} \right] + \left( {P_{GR} \left( h \right)E_{GR} \left( h \right)} \right)} \right\}$$

Here $E_{DGi} \left( h \right)$, $E_{STj} \left( h \right)$, and $E_{GR} \left( h \right)$ denote the amount of pollutants from $i{\text{th}}$ distributed generating unit, $j{\text{th }}$ storage unit, and the market, at hour ${\text{h}}$, in ${\text{kg}}/{\text{MWh}}$ correspondingly.

The emission variables are symbolized as follows^91^:

27 $$E_{DGi} \left( h \right) = {\text{CO}}_{{2_{{DG_{i} }} }} \left( h \right) + {\text{SO}}_{{2_{{DG_{i} }} }} \left( h \right) + {\text{NO}}_{{x_{{DG_{i} }} }} \left( h \right)$$

Here ${\text{CO}}_{{2_{{DG_{i} }} }} \left( h \right)$, ${\text{SO}}_{{2_{{DG_{i} }} }} \left( h \right)$ and ${\text{NO}}_{{x_{{DG_{i} }} }} \left( h \right)$ characterize the emissions of ${\text{CO}}_{2}$, ${\text{SO}}_{2}$, and ${\text{NO}}_{x}$ correspondingly from the $i{\text{th}}$ DG sources during the hour ${\text{h}}$ of the day.

28 $$E_{STj} \left( h \right) = {\text{CO}}_{{2_{{ST_{i} }} }} \left( h \right) + {\text{SO}}_{{2_{{ST_{i} }} }} \left( h \right) + {\text{NO}}_{{x_{{ST_{i} }} }} \left( h \right)$$

Here ${\text{CO}}_{{2_{{ST_{i} }} }} \left( h \right)$, ${\text{SO}}_{{2_{{ST_{i} }} }} \left( h \right)$ and ${\text{NO}}_{{x_{{ST_{i} }} }} \left( h \right)$ signify the emissions of ${\text{CO}}_{2}$, ${\text{SO}}_{2}$, and ${\text{NO}}_{x}$ correspondingly from the $j{\text{th}}$ storage unit at hour ${\text{h}}$.

29 $$E_{GR} \left( h \right) = {\text{CO}}_{{2_{GR} }} \left( h \right) + {\text{SO}}_{{2_{GR} }} \left( h \right) + {\text{NO}}_{{x_{GR} }} \left( h \right)$$

Here ${\text{CO}}_{{2_{GR} }} \left( h \right)$, ${\text{SO}}_{{2_{GR} }} \left( h \right)$ and ${\text{NO}}_{{x_{GR} }} \left( h \right)$ represent the emissions of ${\text{CO}}_{2}$, ${\text{SO}}_{2}$, and ${\text{NO}}_{x}$ correspondingly from the macro-grid or utility during the hour ${\text{h}}$ of the day.

### Constraints and limitations

#### Load-generation balance

30 $$\mathop \sum \limits_{k = 1}^{{N_{KL} }} P_{LLk} \left( h \right) + \mathop \sum \limits_{m = 1}^{{N_{PHEV} }} P_{PHEV,m} \left( h \right) = \mathop \sum \limits_{i = 1}^{{N_{DG} }} \left[ {P_{DGi} \left( h \right)} \right] + \mathop \sum \limits_{j = 1}^{{N_{ST} }} \left[ {P_{STj} \left( h \right)} \right] + \left( {P_{GR} \left( h \right)} \right)$$

Here, $P_{LLk}$ represents the load magnitude of the $k{\text{th}}$ load, whilst $N_{KL}$ denotes the total number of load levels present within the utility, as defined in Ref.^91^.

#### Generated power

The entire set of units, including the market, storage units, and distributed generators (DG), has defined lower and upper limits that regulate their power generation capacities, as described in Ref.^91^. The output power from the MG components should achieve the following constraints^53^.

31 $$\begin{gathered} P_{DGi,min} \left( h \right) \le P_{DGi} \left( h \right) \le P_{DGi,max} \left( h \right) \hfill \\ P_{STj,min} \left( h \right) \le P_{STj} \left( h \right) \le P_{STj,max} \left( h \right) \hfill \\ P_{GR,min} \left( h \right) \le P_{GR} \left( h \right) \le P_{GR,max} \left( h \right) \hfill \\ \end{gathered}$$

The formula provided in Eq. (9) stipulates that the power generated from distributed generation (DG), battery, and grid sources must fall within their designated minimum and maximum limits, denoted by “min” and “max” respectively.

#### DGs’ ramp rate constraints

This constraint pertains to the adjustment of the output power from distributed generators (DGs), describing the condition as follows^78^:

32 $$R_{down}^{i} \cdot \Delta h \le P\left( h \right)^{i} - P\left( {h - 1} \right)^{i} \le R_{up}^{i} \cdot \Delta h$$

where $R_{down}^{i}$ and $R_{up}^{i}$ are the ramp-down and ramp-up of the $i{\text{th}}$ DG output power, respectively, and $\Delta h$ is the time step in hours.

#### Battery charging/discharging states

To avoid the damage of the battery, the following constraint should be achieved^78,95^:

33 $$E_{b}^{min} \le E_{b} \left( h \right) \le E_{b}^{max}$$

34 $$P_{ch} \left( h \right) \le P_{ch}^{rated} ,P_{disch} \left( h \right) \le P_{disch}^{rated}$$

where $E_{b}^{min}$ represents the minimum stored energy in the battery while $E_{b}^{max}$ denotes the maximum stored energy, $P_{ch}^{rated}$ is the battery rated charge power, and $P_{disch}^{rated}$ represents the battery rated discharge power during each time interval $\Delta h$.

The battery stored energy can be calculated as follows^78^:

35 $$E_{b} \left( h \right) = E_{b} \left( {h - 1} \right) + \xi_{ch} P_{ch} \left( h \right)\Delta h - \frac{1}{{\xi_{disch} }}P_{disch} \left( t \right)\Delta h$$

where $\xi_{ch}$ is the charging efficiency while $\xi_{disch}$ represents the discharging efficiency.

Here $E_{b} \left( h \right)$ and $E_{b} \left( {h - 1} \right)$ represent the energy stored in the battery at hours $h$ and $h - 1$, respectively. $P_{ch}$ is the permissible charging rate, while $P_{disch}$ is the permissible discharging rate during a specific time interval ($\Delta h$). The battery’s charging and discharging efficiency are denoted by $\xi_{ch}$ and $\xi_{disch}$ respectively^96,97^.

### Formulation of multi-objective energy management problem

The multi-objective energy management problem is formulated as follows:

36 $$Minimize f_{CE} \left( {f_{T.C } ,f_{T.E} } \right)$$

In this context, $f_{T.C }$ represents the objective function focused on cost minimization, while $f_{T.E}$ is the objective function targeting emissions reduction. By integrating a price penalty factor (ρ), the multi-objective energy management problem can be transformed into a single-objective optimization problem, as shown in Eq. (26). The approach for determining the value of ρ is detailed in^98^.

37 $$Minimize f_{CE} = \mathop \sum \limits_{i = 1}^{N} \left( { \left( {w \times f_{T.C } } \right) + \left( {\rho \times \left( {1 - w} \right) \times f_{T.E} } \right)} \right)$$

In this context, the weighting factor ‘$w$’ signifies the degree of importance assigned to a specific objective function. With ‘$w$’ set to 1, the optimization primarily emphasizes the reduction of operational costs. Conversely, assigning ‘$w$’ a value of 0 prioritizes the minimization of emissions. In the context of multi-objective energy management, the ‘$w$’ value is systematically reduced from 1 to 0, and at each decrement, a compromised solution is generated. Ultimately, the best compromised solution (BCS) is determined using the fuzzy membership approach outlined in section “Fuzzy logic-based selection of optimal compromise solution”, where a decrease in ‘$w$’ leads to a simultaneous increase in operational costs and a reduction in pollutant emissions.

## Fuzzy logic-based selection of optimal compromise solution

Before making a decision, it is crucial to determine the optimal compromise solution from the available set of optimal solutions^99,100^. To identify the best compromise solution, the author employed a fuzzy membership approach^101^. In jth objective function,$f_{j }$ of individual k is characterized by a membership function $\mu_{j }^{k}$ due to indefinite characteristic of decision maker’s conclusion which is represented as follows^102^:

38 $$\mu_{j}^{k} = \left\{ {\begin{array}{*{20}l} 1 \hfill & { f_{j } \le f_{j}^{min} } \hfill \\ {\frac{{f_{j}^{max} - f_{j} }}{{f_{j}^{max} - f_{j}^{min} }}} \hfill & {f_{j}^{min} < f_{j} < f_{j}^{max} } \hfill \\ 0 \hfill & { f_{j} \ge f_{j}^{max} } \hfill \\ \end{array} } \right.$$

where $f_{j}^{max}$ denote the maximum value of jth fitness function while the latter’s minimum value is represented by $f_{j}^{min}$ in the pool of non-dominated solutions. Here, the normalized membership function $\mu^{k}$ is also determined accordingly for each non-dominated solution k as follows^103^:

39 $$\mu^{k} = \frac{{\mathop \sum \nolimits_{j = 1}^{N} \,\mu_{j}^{k} }}{{\mathop \sum \nolimits_{k = 1}^{r} \mathop \sum \nolimits_{j = 1}^{N} \mu_{j}^{k} }}$$

Here, the overall number of non-dominated solutions is denoted by r. The best compromise solution is composed of maximum value, $\mu^{k} .$

## Uncertainty models for Wind and Solar Energy

Different types of PDFs (Probability Density Function) have been deployed for the characterization of stochastic output power from the RESs. The wind turbine-based power relies upon the speed of the wind. As per the literature^104–106^, Weibull PDF forms the basis for wind speed probability.

40 $$f_{wv} \left( v \right) = \left( {\frac{\alpha }{\lambda }} \right)\left( {\frac{v}{\lambda }} \right)^{{\left( {\alpha - 1} \right)}} exp[^{{ - \left( {\frac{v}{\lambda }} \right)}} ]^{\alpha } \,\,\,\,for\,\,\,\, 0 < v < \infty$$

Here, the shape parameter of Weibull PDF is denoted by $\alpha$ whereas $\lambda$ corresponds to the scale of Weibull PDF. These variable values are sourced from the literature^104^. The following Eq. (41) shows the average of Weibull PDF.

41 $$M_{w} = \lambda *\Gamma \left( {1 + \alpha^{ - 1} } \right)$$

The equation given below (25) describes the $\Gamma$ function.

42 $$\Gamma \left( {x^{\prime } } \right) = \mathop \smallint \limits_{0}^{\infty } e^{ - t} t^{{x^{\prime } - 1}} dt$$

**Figure 2:**
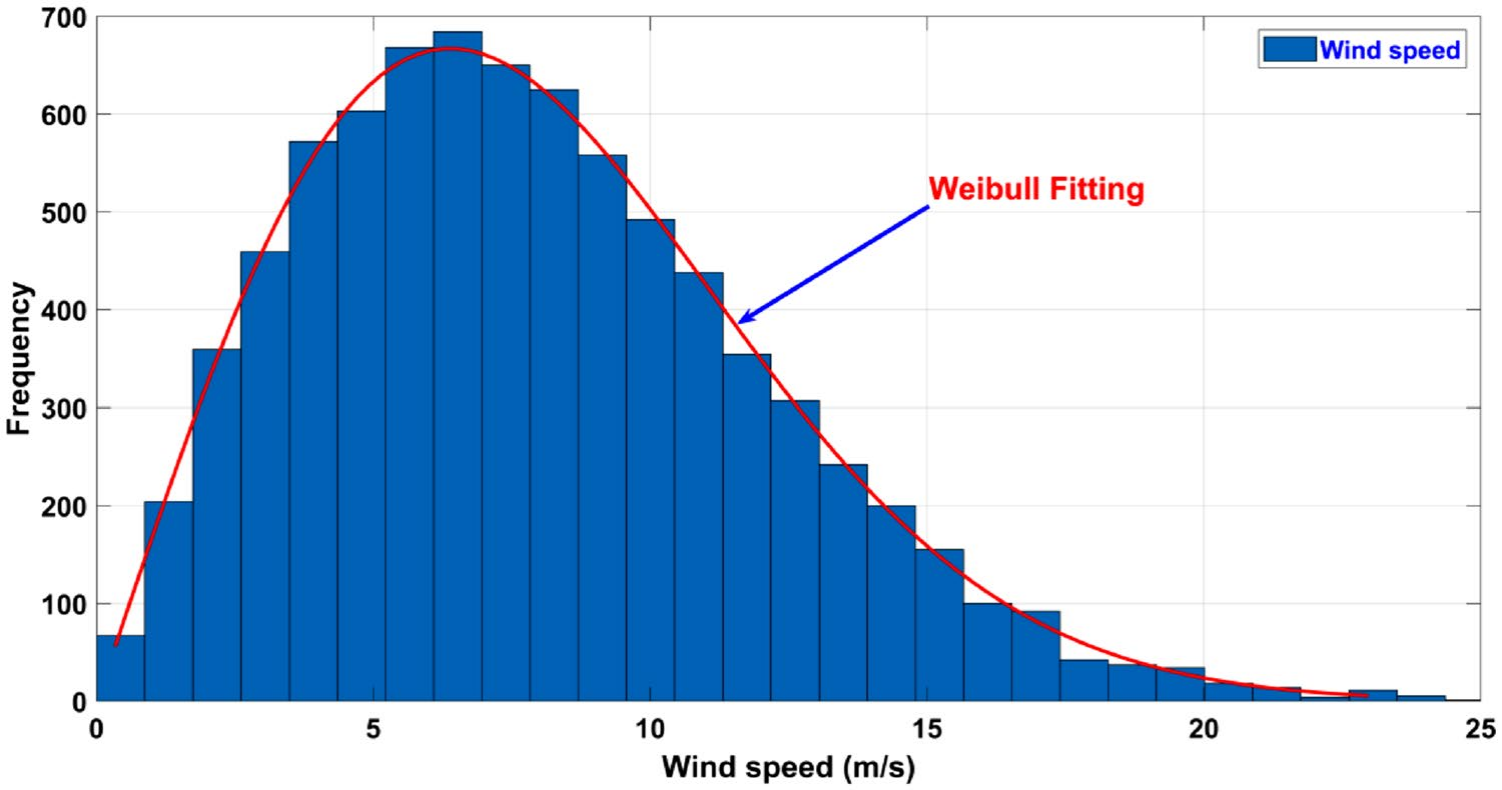
Wind speed variation for wind farm.

The fluctuations that occur in wind speed for the wind farm are shown in Fig. 2. As per the literature^7^, both scale and shape parameter values are decided. On the other hand, the PDF parameter values are chosen according to the study conducted earlier^104,107^. The aggregated rated output generated by the wind farm with capacity of 15 MW is achieved by connecting 5 wind generators in the considered microgrid test system. Each individual wind generator has a capacity of 3 MW. The subsequent Eq. (43) describes the power produced by the wind generators that relies upon the speed of the wind.

43 $$P_{WG} = \begin{array}{*{20}l} 0 \hfill & {for} \hfill & {v \le v_{in} } \hfill \\ {P_{{W_{r} }} \left( {\frac{{v - v_{in} }}{{v - v_{out} }}} \right)} \hfill & {for} \hfill & {v_{in} \le v \le v_{r} } \hfill \\ {P_{{W_{r} }} } \hfill & {for} \hfill & {v_{r} \le v \le v_{out} } \hfill \\ \end{array}$$

Here, $P_{{W_{r} }}$ corresponds to a single turbine’s rated power whereas the cut-in speed is denoted by $v_{in}$. On the other hand, the cut-out speed is characterized by $v_{out}$ and the rated speed is denoted by $v_{r}$. In this research work, various Weibull parameters are considered for the distribution of wind speed in line with literature^104,107^. From the wind generators, rated power is generated within the wind speed range that falls between the cut-in and cut-out thresholds. As per the literature^104,107^, the probability of such discrete zones is shown in the Eq. (44).

44 $$f_{{P_{WG} }} = 1 - {\text{exp}}\left[ { - \left( {\frac{{v_{in} }}{\lambda })^{\alpha } } \right] + {\text{exp}}} \right[ - \left( {\frac{{v_{out} }}{\lambda })^{\alpha } } \right]\,\,\,\,for\,\,\,\, \left( {P_{WG} = 0} \right)$$

45 $$f_{{P_{WG} }} = {\text{exp}}\left[ { - \left( {\frac{{v_{r} }}{\lambda })^{\alpha } } \right] - {\text{exp}}} \right[ - \left( {\frac{{v_{out} }}{\lambda })^{\alpha } } \right]\,\,\,\,for \,\,\,\,\left( {P_{WG} = P_{WR } } \right)$$

In continuous region, the probability distribution for wind power is as follows.

46 $$f_{{P_{W G} }} = \frac{{\alpha \left( {v_{r} - v_{in} } \right)}}{{\lambda^{\alpha } *P_{wr} }}\left[ {v_{in} + \frac{{P_{WG } }}{{P_{wr} }}\left( {v_{r} - v_{in} } \right)} \right]^{\alpha - 1} exp - \left( {\frac{{(v_{in} + \frac{{P_{WG } }}{{P_{wr} }}\left( {v_{r} - v_{in} } \right)}}{\lambda }} \right)^{\alpha }$$

Likewise, the power generated by solar PV system completely relies upon the solar irradiance (G) that suits the guidelines as per lognormal PDF^104,107^. As per the literature^108^, the following equation shows the probability of solar irradiance with mean as well as standard deviation.

47 $$f_{PV} \left( G \right) = \frac{1}{{G\sigma \sqrt {2\pi } }}\exp \left[ { - \frac{{\left( {\ln x - \mu } \right)^{2} }}{{2\sigma^{2} }}} \right] \,\,\,\,for\,\,\,\, G > 0$$

The subsequent Eq. (48) yields the mean of lognormal distribution ($M_{Lgn}$)

48 $$M_{Lgn} = \exp \left( {\mu + \frac{{\sigma^{2} }}{2}} \right)$$

**Figure 3:**
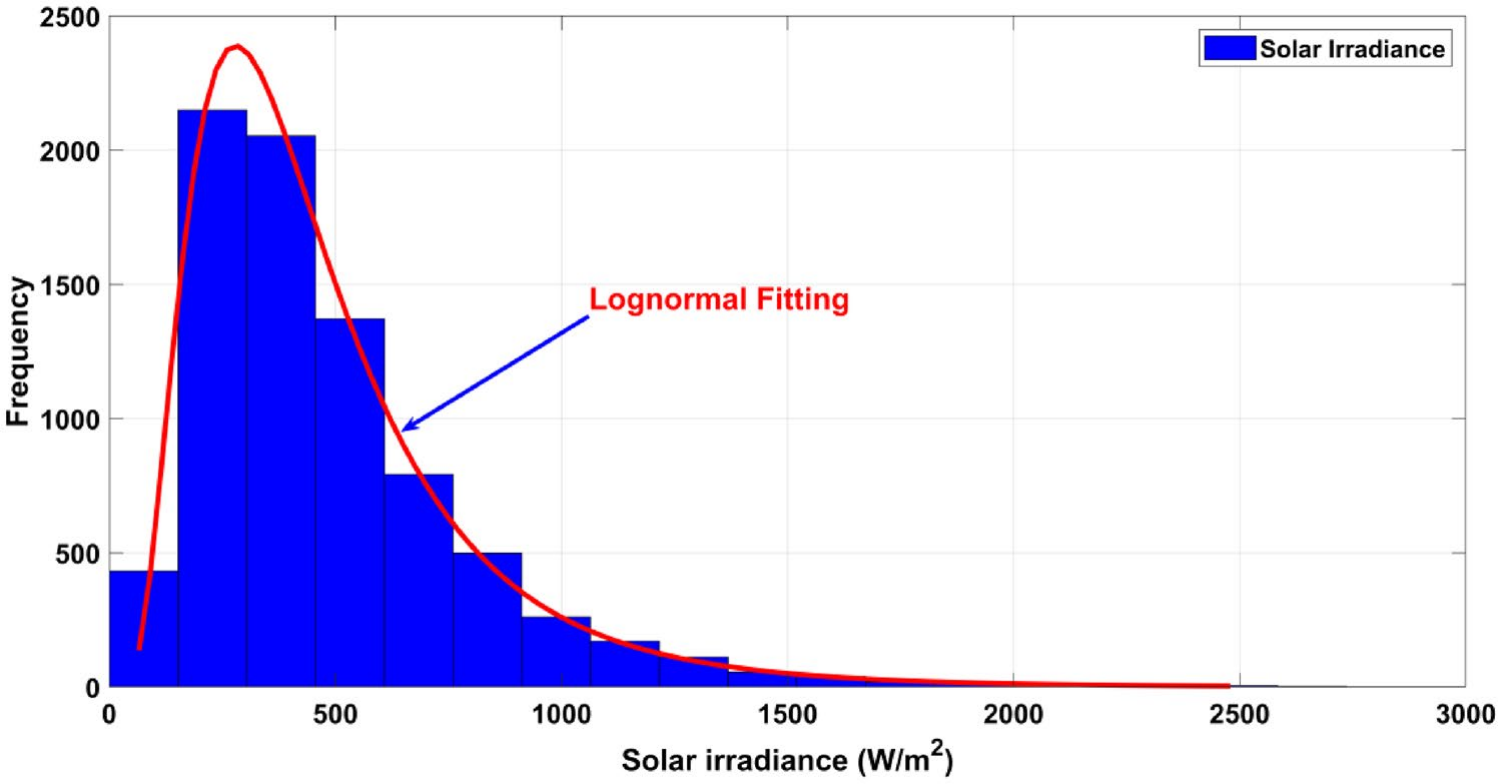
Solar irradiance distribution for solar PV.

Figure 3 shows the frequency distribution for lognormal fitting and solar irradiance in case of simulating the Monte-Carlo scenario using 8,000 samples. The solar PV output power is expressed herewith.

49 $$P_{PV} \left( G \right) = \begin{array}{*{20}l} {P_{PVr} \left( {\frac{{G^{2} }}{{G_{std} R_{C} }}} \right)} \hfill & {for} \hfill & { 0 \le G \le R_{C} } \hfill \\ {P_{PVr} \left( {\frac{G}{{G_{std} }}} \right) } \hfill & {for} \hfill & { G \ge R_{C} } \hfill \\ \end{array}$$

In a standard environmental setting, solar irradiance is denoted as $G_{std}$, with specific irradiance represented by $R_{C}$. For $G_{std}$, the value assumed is 800 ${\text{W}}/{\text{m}}^{2}$, while $R_{C}$ is set at 120 ${\text{W}}/{\text{m}}^{2}$. For the PV module, the rated output power $P_{PVr}$ is specified as 25 MW.

## Modeling of microgrid

Utilizing individual distributed generators (DGs) can introduce numerous challenges, highlighting the importance of adopting a system approach. This approach treats generation and associated loads as a subsystem or microgrid^109,110^. By aggregating distributed generators (DGs) within a microgrid and harnessing renewable energies in large quantities, various issues related to economy, technology, and environment can be carefully studied within the target system, enabling informed decisions for improved operational management^111,112^. Furthermore, distributed generation encompasses a diverse array of prime mover technologies, including internal combustion (IC) engines, gas turbines, microturbines, photovoltaic systems, fuel cells, and wind power. These emerging technologies typically exhibit lower emissions and have the potential to achieve lower costs, thus challenging traditional economies of scale^78^. For instance, fuel cells, which generate electricity from hydrogen and oxygen, primarily emit water vapor. However, during the reformation of natural gas or other fuels, they may produce some $NO_{X}$ and $CO_{2}$ emissions^113,114^. Despite their higher initial costs, fuel cells are generally more efficient and have lower emissions compared to microturbines. In this paper, a typical low-voltage (LV) microgrid is considered, incorporating various DGs such as microturbines (MT), low-temperature fuel cells (PAFC), photovoltaic (PV) arrays, wind turbines (WT), and storage devices like lead-acid batteries^78^. It is assumed that all DG sources generate active power at unity power factor without requesting or producing reactive power. Additionally, a power exchange link exists between the microgrid and the utility (LV network), facilitating energy trading throughout various hours of the day based on decisions made by the microgrid central controller (MGCC).

## Results and discussion

The microgrid test system under examination comprises a distributor and various distributed generators (DGs), including photovoltaic panels (PV), wind turbines (WT), microturbines (MT), fuel cells (FC), and batteries^92^. In the proposed model, the objective function aggregates the total cost of the microgrid, encompassing power generation costs and startup/shutdown costs of units, in addition to the net emission of pollutants. This problem is addressed through three distinct scenarios. The primary case, where all units are dispatched based on their actual constraints. In the second scenario, both the wind turbine (WT) and solar photovoltaic (PV) systems operate at their maximum output levels. In the third scenario, the utility is treated as an unbounded unit that can exchange energy with the microgrid without any constraints. The total load demand within the microgrid for a typical day includes primarily residential areas, one industrial feeder serving a small workshop, and one feeder with light commercial consumers, as documented in Ref.^92^. The cumulative energy demand for the specified day amounts to 1695 kilowatt-hours (kWh). Furthermore, the study takes into account the real-time variation in energy prices in the market for the specified day, as documented in the earlier study^92^. To ensure the flexible operation of the microgrid, the optimization algorithm dynamically assigns “on” or “off” states to three distributed generation (DG) units—MT (Micro Turbine), PV (Photovoltaic), and WT (Wind Turbine)—during the power dispatch problem, considering both objectives. Similarly, since the microgrid operates in grid-connected mode, the utility is consistently set to the “on” state. In order to comprehensively evaluate the impact of the battery and PAFC (Proton Exchange Membrane Fuel Cell) on grid operation and to maximize the benefits of these resources, the “on” state is deliberately chosen for these respective units. The minimum and maximum generation limits of the DG sources are obtained from Ref.^92^. Furthermore, the bid coefficients in cents of Euro (€ct) per kWh, as well as emissions in kilograms per MWh, assumed by the DG sources, are extracted from Ref.^92^. To streamline our analysis, all units under consideration in this research study are assumed to operate exclusively in electricity mode, without requiring heat during the analyzed period. It’s important to highlight that the enhanced integration of renewable energies stands as a key motivation behind micro-grid installations. In actual micro-grid operations, forecasts of future requirements are crucial for preparing flexible systems to respond appropriately. Although renewable energy may not follow traditional operational patterns, its behavior can be anticipated, and forecast information becomes crucial for optimizing system efficiency within microgrids. In this study, the power output of photovoltaic (PV) and wind turbine (WT) units is projected using an expert prediction model. However, this aspect falls beyond the scope of the current paper and will be addressed in future research. Table 3 provides an overview of the forecasted output of these units. The maximum allowable daily power extracted from the PV and WT are taken from the earlier study^92^. The daily load power and the energy market price in the typical micro-grid considered are taken from Ref.^92^.

**Table 3:** Predicted values of WT and PV^92^.

Hour	WT(kW)	PV(kW)	Load (kW)	Electrical energy price €ct/kWh
1	1.7850	0	52.00	0.2300
2	1.7850	0	50.00	0.1900
3	1.7850	0	50.00	0.1400
4	1.7850	0	51.00	0.1200
5	1.7850	0	56.00	0.1200
6	0.9150	0	63.00	0.2000
7	1.7850	0	70.00	0.2300
8	1.3050	0.200	75.00	0.3800
9	1.7850	3.750	76.00	2.5000
10	3.0900	7.525	80.00	4.0000
11	8.7750	10.45	78.00	4.0000
12	10.410	11.95	74.00	4.0000
13	3.9150	23.90	72.00	1.5000
14	2.3700	21.05	72.00	4.0000
15	1.7850	7.875	76.00	2.0000
16	1.3050	4.225	80.00	1.9500
17	1.7850	0.550	85.00	0.6000
18	1.7850	0	88.00	0.4100
19	1.3020	0	90.00	0.3500
20	1.7850	0	87.00	0.4300
21	1.3005	0	78.00	1.1700
22	1.3005	0	71.00	0.5400
23	0.9150	0	65.00	0.3000
24	0.6150	0	56.00	0.2600

To assess the effectiveness of the suggested SaCryStAl technique, a simulation is conducted comprising 50 trial runs aimed at minimizing operating costs. The controlling parameters of the proposed algorithm are selected as population size of 30 and maximum iteration of 1000^78^. The input data, including bids, technical coefficients, and emission coefficients of the DG sources for the microgrid test system under consideration, are sourced from Ref.^92^. Five distributed generation (DG) sources with associated characteristics generate electricity within the micro-grid. Any excess or shortfall of energy within the grid is balanced through exchange with the utility at the point of common coupling. All units, including the utility sourced from the macro grid, are obliged to operate within their power limits while meeting specified constraints. The output power levels of the wind turbine and solar cell based on the predicted values are presented in Table 3^92^.

### Case-I: operation of distributed energy sources within prescribed bounds

The first test case analyzed in this study entails operating all distributed generators (DGs) and the grid within predefined constraints, as detailed in Table 4. Furthermore, Table 4 illustrates the optimal generation schedule for 24 h aimed at minimizing both cost and emissions. It is evident from Table 4 that, following the new approach, a substantial portion of the load is initially supplied by the fuel cell within the grid and utility via the point of common coupling during the early hours of the day. This preference is due to the lower bids of these units compared to others during this timeframe. As demand and utility bids rise in subsequent hours, distributed generation (DG) units adjust their output levels based on priority, prioritizing lower costs and emissions accordingly. Consequently, DG units start up sequentially as requested by the micro-grid regulatory controller and energy import from the macro grid is replaced by export actions to enhance revenue and reduce net emissions during this period. Additionally, it’s worth noting that battery charging occurs during the early hours when prices are low, while discharge actions are postponed to midday when the load curve peaks. Furthermore, leveraging renewable energy sources such as wind and solar reduces pollution but may increase the operating cost. Hence, the utilization of energy from these resources should be constrained, taking into account emission and economic factors.

**Table 4:** Optimal generation schedule for minimization of operating cost and emission (Case-I).

Hour	$P_{d}$(kW)	$P_{pv}$(kW)	$P_{wt}$(kW)	$P_{mt}$(kW)	$P_{FC}$(kW)	$P_{Batt}$(kW)	$P_{grid}$(kW)
1	52	0	0	13.87	25.32	− 4.52	17.32
2	50	0	0	17.33	22.83	10.25	− 0.41
3	50	0	0	11.92	26.11	0.42	11.55
4	51	0	0	7.89	20.86	− 0.63	22.87
5	56	0	0	20.93	20.25	5.91	8.91
6	63	0	0	12.84	29.55	4.59	16.02
7	70	0	0	24.83	30	9.54	5.63
8	75	0	0	20.32	27.95	11.82	14.91
9	76	0	1.57	23.78	30	13.99	6.66
10	80	12.13	3.24	30	30	27.84	− 23.21
11	78	12.13	8.42	27.45	30	30	− 30
12	74	4.51	11.22	27.33	30	29.91	− 28.97
13	72	1.24	1.63	28.57	29.78	28.21	− 17.43
14	72	7.85	4.16	30	29.98	30	− 29.99
15	76	1.45	2.33	28.31	30	30	− 16.09
16	80	1.15	0.55	28.31	28.18	28.36	− 6.55
17	85	2.31	0	20.23	30	19.13	13.33
18	88	0	0	22.35	30	11.43	24.22
19	90	0	0	21.14	25.87	21.17	21.82
20	87	0	0	24.97	29.98	4.93	27.12
21	78	0	0	21.32	29.92	30	− 3.24
22	71	0	0	23.14	27.46	24.69	− 4.29
23	52	0	0	14.56	20.45	4.12	12.87
24	50	0	0	20.05	19.32	4.51	6.12

**Figure 4:**
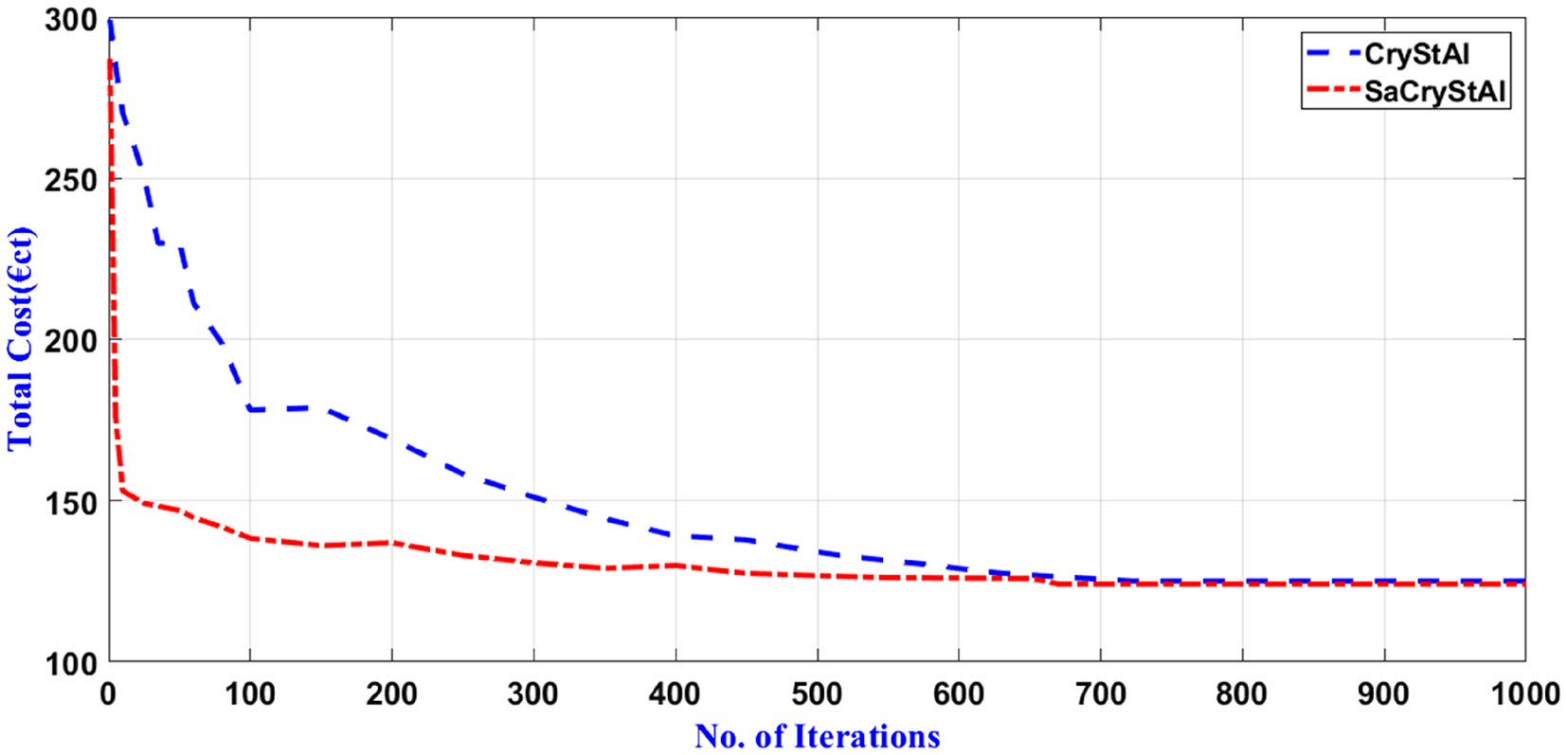
Convergence characteristic for the minimization of operating cost (Case-I).

**Figure 5:**
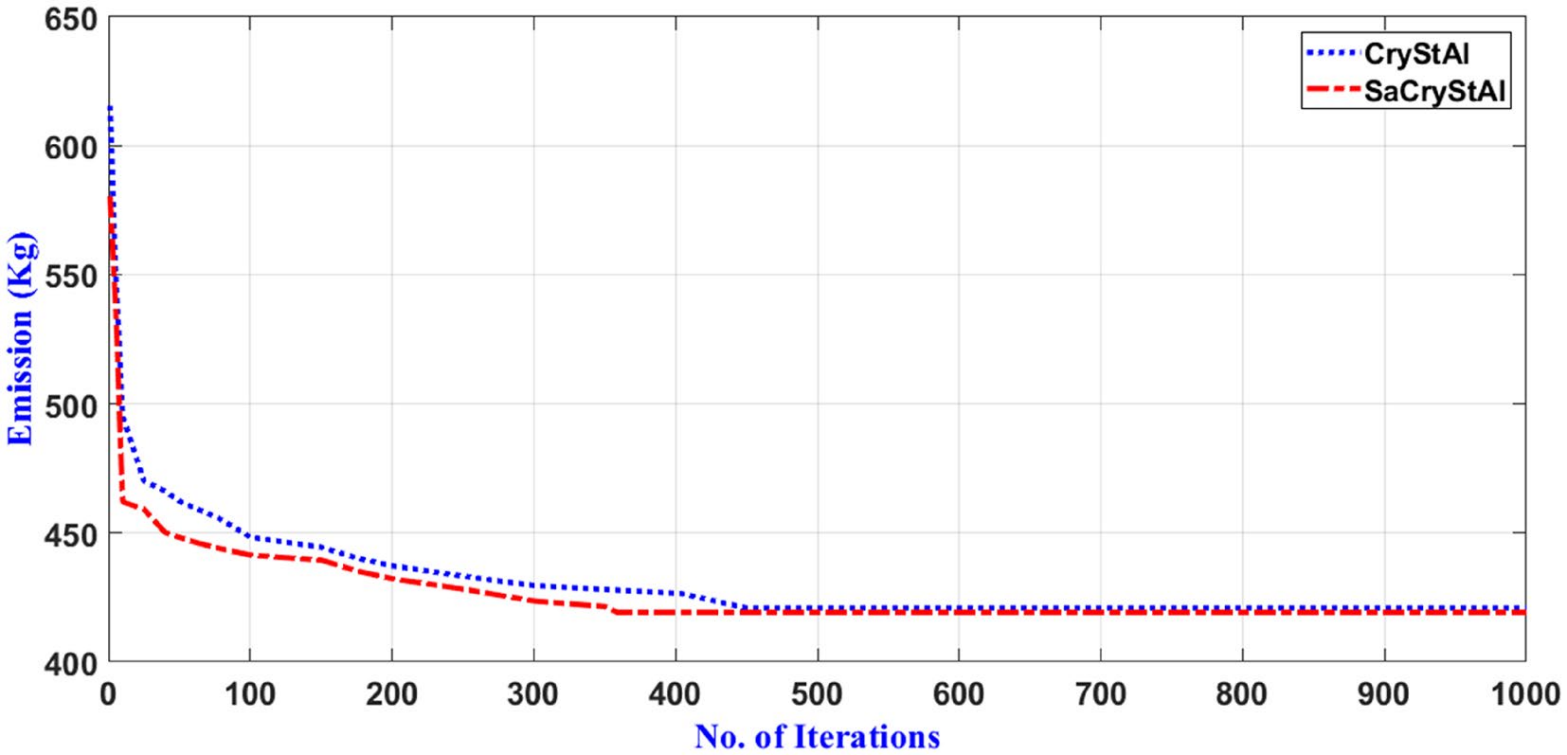
Convergence profile for emission minimization (Case-I).

**Figure 6:**
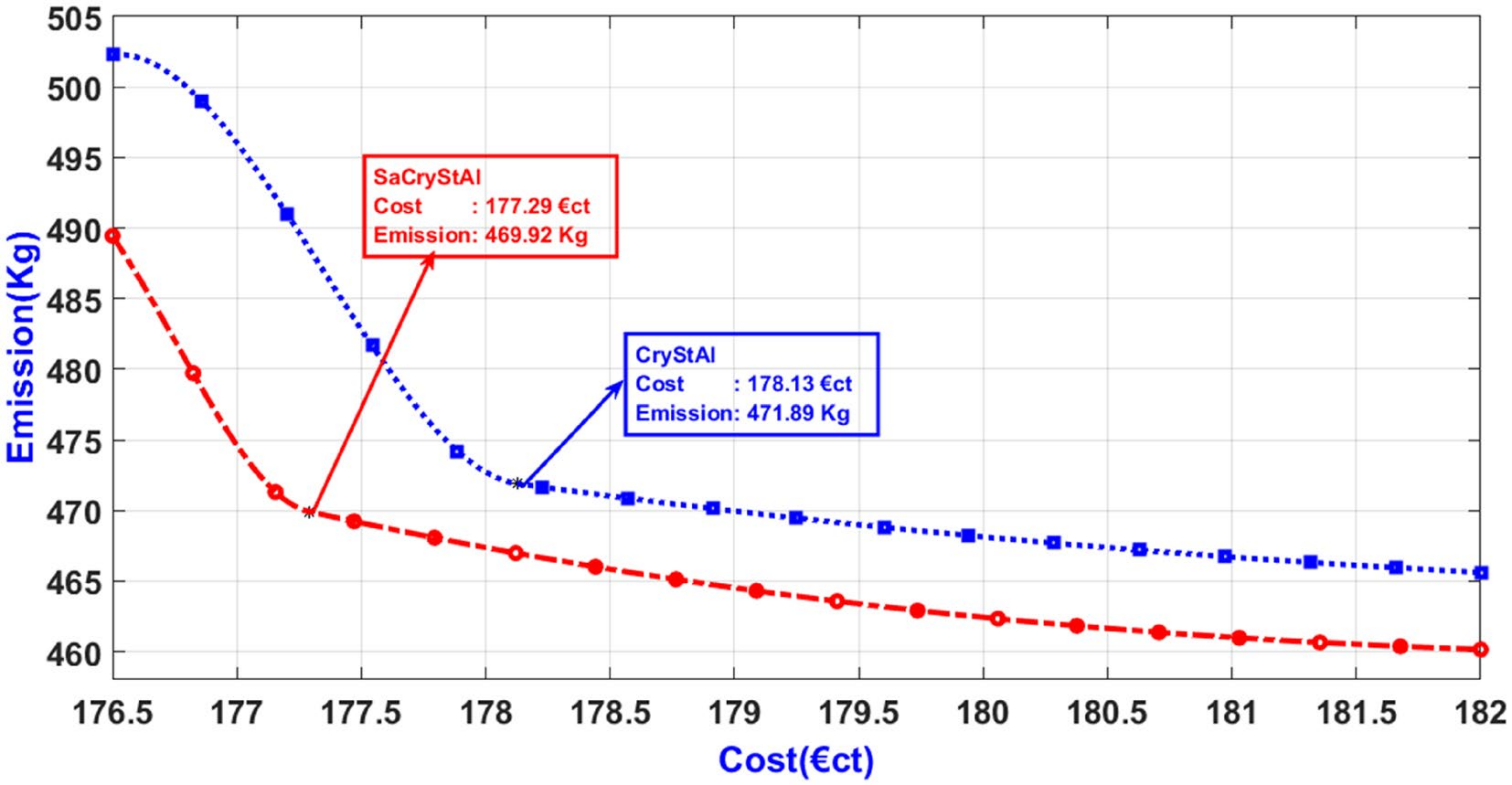
Trade-off characteristic between emissions and costs (Case-I).

Tables 5 and 6 present the statistical outcomes of optimization algorithms, along with a concise comparison of their performances in the primary scenario. When evaluating performances based on both the best and worst solutions for cost and emission objectives, it becomes evident that the proposed optimization algorithm not only delivers superior outcomes but also demonstrates faster convergence. Additionally, statistical indices such as average and standard deviation further validate the algorithm’s advantage in the optimization process. Tables 5 and 6 showcase standard deviation values for cost and emission objectives with the new algorithm limited to 0.006 and 0.005, respectively, indicating the excellent performance of the proposed model. By incorporating an oppositional population mechanism during the optimization process, the proposed algorithm explores further enhancements in both performance characteristics and optimal solutions. To provide a deeper insight into SaCryStAl’s performance, the convergence characteristics of SaCryStAl and CryStAl algorithms for the best solution and each single objective are separately illustrated in Figs. 2 and 3. The operation cost was minimized by assigning the weighting factor $w$ as unity. The proposed algorithm provides the least operation cost of 124.15 €ct compared with CryStAl, FSAPSO^91^, GA^91^, GWO^91^, PSO^91^, WOA^91^, and KH^91^. The optimization findings indicate a close alignment between the minimum operating cost and its mean value, underscoring the precision of the proposed algorithm. By adjusting the weighting factor from 1 to 0, we achieved an optimal emission value of 419.14 kg. Figures 4 and 5 depict that the cost objective function reaches its minimum after around 670 iterations with the new method and remains stable thereafter, contrasting with the CryStAl algorithm, which converges in approximately 690 iterations. Similarly, the emission objective function reaches its minimum after about 428 iterations with the new method, while the CryStAl algorithm converges in about 417 iterations. Additionally, Fig. 6 highlights the superior performance of all mentioned algorithms when considering both objectives. Employing a fuzzy logic approach enabled the proposed algorithm to achieve the global best compromise solution for both generations cost and emission minimization.

**Table 5:** Statistical comparative results with other algorithms for minimization of operating cost (Case-I).

Algorithm	Mean (€ct)	Standard deviation (€ct)	Max (€ct)	Min (€ct)	CPU time (s)
SaCryStAl	126.89	0.003	147.83	124.15	97.19
CryStAl	127.72	0.005	149.16	125.03	97.32
FSAPSO^91^	125.91	0.006	125.92	125.91	NA
GA^91^	151.89	36.23	210.46	125.91	114.67
GWO^91^	151.57	40.20	824.30	128.93	132.17
PSO^91^	145.28	53.52	830.83	126.16	556.64
WOA^91^	129.05	8.99	307.55	126.09	149.17
KH^91^	148.57	0.009	1337.7	105.94	104.17

**Table 6:** Statistical comparison of results with other algorithms for emission minimization (Case-I).

Algorithm	Mean (kg)	Standard deviation (kg)	Max (kg)	Min (kg)	CPU time (s)
SaCryStAl	420.96	0.002	428.04	419.14	76.41
CryStAl	422.03	0.004	429.25	420.89	76.83
FSAPSO^91^	422.02	0.005	422.03	422.02	NA
GA^91^	506.78	89.25	680.33	422.02	119.85
GWO^91^	580.88	300.97	2699.2	451.54	167.44
PSO^91^	500.44	216.42	2943.4	425.43	523.87
WOA^91^	428.79	68.54	2135.3	423.25	124.92
KH^91^	436.59	0.004	118.97	420.57	79.41

Figure 6 depicts the Pareto fronts of the respective trade-off objectives obtained from various comparison methods, alongside the best compromise solutions. Additionally, the distribution of non-dominated solutions along the Pareto optimal front, as observed in Fig. 6, validates the effectiveness of the proposed algorithm in addressing nonlinear multi-objective optimization problems. Moreover, the computational time for both operating cost and emission minimization using the proposed algorithm is notably shorter, indicating the high solution quality achieved. Overall, the optimization results strongly support the proposed algorithm’s capability to address challenges related to equality and inequality in energy management problems.

### Case-II: operation of microgrid with rated wind power

**Figure 7:**
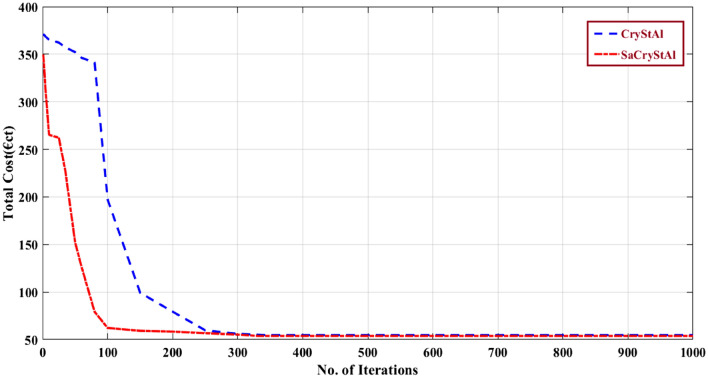
Convergence characteristic for the minimization of operating cost (Case-II).

**Figure 8:**
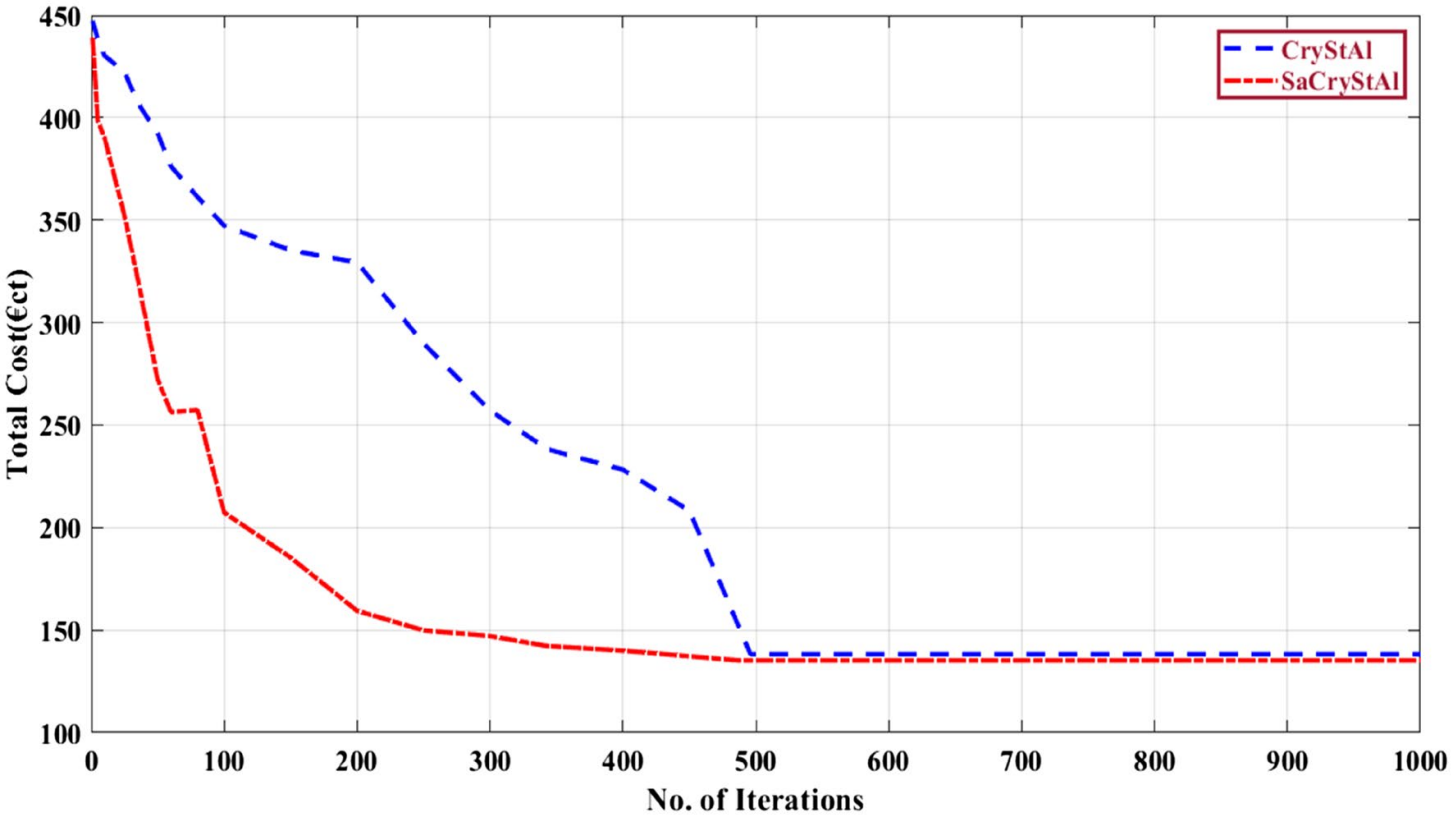
Convergence profile for emission minimization (Case-II).

**Figure 9:**
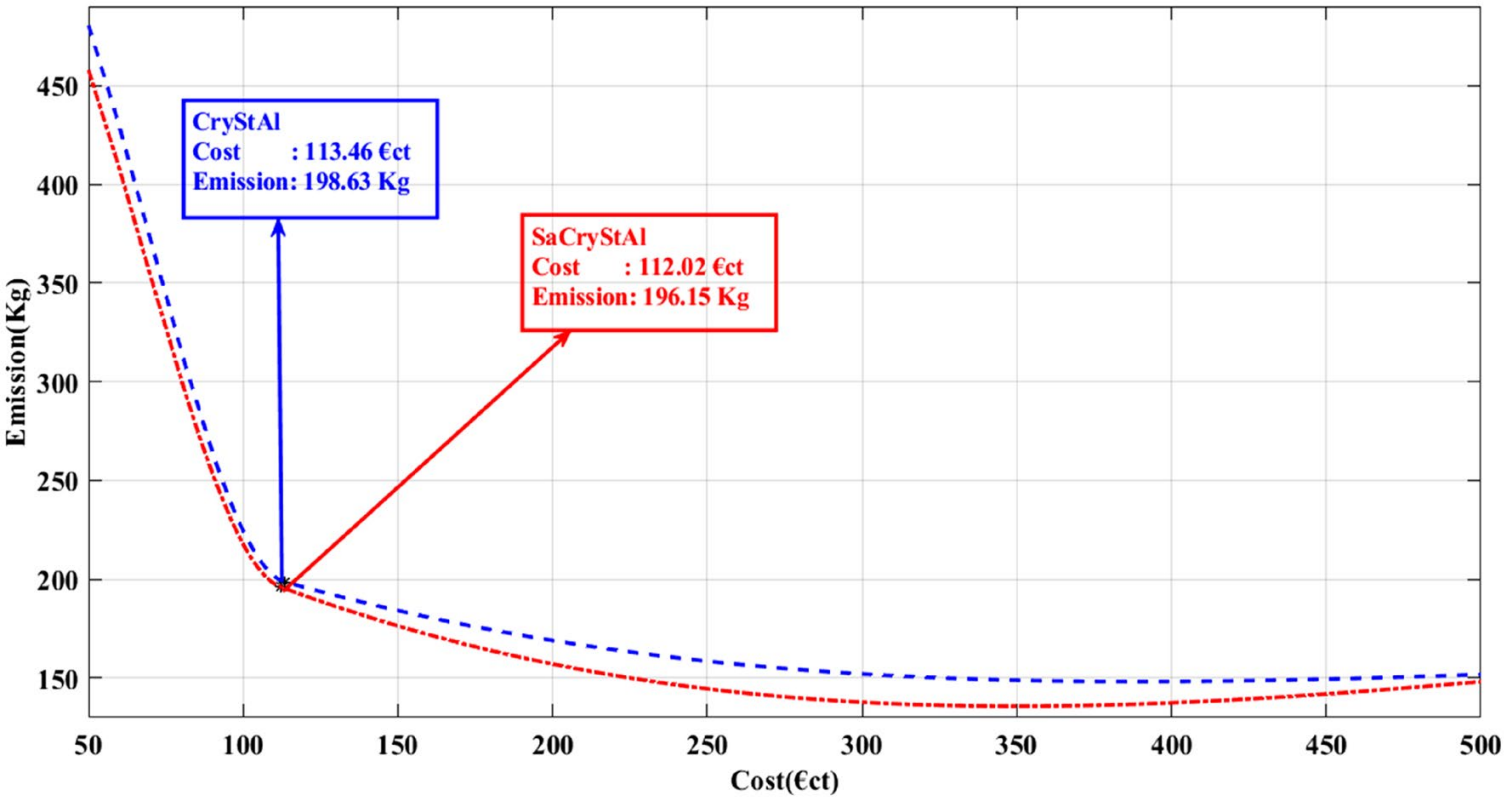
Trade-off characteristic between emissions and costs (Case-II).

The WT is run at its rated power of 15 kW in the second scenario that is taken into consideration^115^. In this scenario, the proposed SaCryStAl is used to distribute the load to the MG components. While the PV generation is nil and the battery is in the charging stage, the MT, WT, FC, and grid actively contribute to meeting the electrical load during the first eight hours. The PV began to share the load with the other mounted devices during the second interval. In this instance, extra electricity is sold to the grid. The load is supported during the last hours by WT, MT, FC, and battery^115^. Table 7 lists the best outcomes and statistical variables that were taken into account in this situation. Setting the weighting factor to 1 prioritizes the minimization of operating costs. Figure 7 illustrates the convergence characteristics achieved by SaCryStAl and CryStAl for operating cost minimization. To minimize emissions, the weighting factor ‘w’ is set to 0, as shown in Fig. 8. SaCryStAl achieved the lowest operating cost of 53.92 €ct, while CryStAl yielded the maximum of 371.28 €ct. SaCryStAl also demonstrated superior variance, standard deviation, and elapsed time. In terms of emissions, SaCryStAl produced the least at 135.186 kg, whereas CryStAl reached a maximum of 439.0481 kg. Detailed results are summarized in Tables 8 and 9. Decreasing the weighting factor ‘w’ from 1 to 0 in steps of 0.001 generates compromise solutions where operating costs increase and emissions decrease simultaneously. Tables 8 and 9 provide a statistical comparison of optimization results for operating cost and emissions, respectively. Through a fuzzy logic approach, the proposed algorithm achieved the global best compromise solution for both objectives. Figure 9 illustrates the trade-off relationship between operating cost and emissions achieved through the utilization of SaCryStAl and CryStAl algorithms. Table 10 presents the comparison of best comparison solution obtained using SaCryStAl, CryStAl and other optimization algorithms for Case I and II. The proposed SaCryStAl algorithm provided a better optimal solution compared to CryStAl. This aims to excel, particularly with the distinctly differentiated Pareto front achieved by SaCryStAl for complex nonlinear optimization problems. In terms of fitness value variation, the SaCryStAl performed well in both goals since it quickly arrived at the best answer. Additionally, SaCryStAl exhibited shorter computational times compared to other optimization algorithms. The optimization results strongly support SaCryStAl’s capability to address the complexities of both equality and inequality present in microgrid energy management challenges, particularly with the incorporation of RES and PHEVs. The obtained simulation results showed excellent performance when WT was operating at its rated power.

**Table 7:** Optimal generation schedule for minimization of operating cost and emission (Case-II).

Hour	$P_{d}$(kW)	$P_{pv}$(kW)	$P_{wt}$(kW)	$P_{mt}$(kW)	$P_{FC}$(kW)	$P_{Batt}$(kW)	$P_{grid}$(kW)
1	52	0	15	24.0394	7.4902	− 24.5295	30
2	50	0	15	12.9382	22.0562	− 29.9921	30
3	50	0	15	29.984	19.05328	− 30	15.9641
4	51	0	15	22.1806	14.9835	− 30	28.8297
5	56	0	15	29.9752	12.6371	− 30	28.3875
6	63	0	15	29.9806	29.9812	− 30	18.0382
7	70	0	15	29.9997	24.9988	− 30	30
8	75	0.263	15	29.9542	29.9732	− 30	29.813
9	76	3.26	15	27.5054	29.9239	30	− 29.6893
10	80	7.603	15	29.9231	4.7821	30	− 7.3029
11	78	11.289	15	29.8834	29.2381	16.8032	− 24.2131
12	74	14.093	15	12.0971	30	30	− 27.1892
13	72	24.752	15	29.9837	30	2.2645	− 30
14	72	23.198	15	10.2093	15.3921	18.9835	− 10.7824
15	76	8.0732	15	27.3891	22.0891	30	− 27.5498
16	80	6.309	15	29.6034	29.1138	29.7023	− 29.7213
17	85	1.752	15	29.5402	30	30	− 21.2874
18	88	0	15	29.7835	30	30	− 16.7832
19	90	0	15	22.6131	30	− 15	22.3891
20	87	0	15	28.0372	30	30	− 16.0348
21	78	0	15	29.6608	30	30	− 26.6608
22	71	0	15	30	30	25.9874	− 29.9874
23	65	0	15	30	20	− 29.9752	29.9752
24	56	0	15	30	12.9098	28.0733	− 29.9823

**Table 8:** Statistical analysis of optimization results for operating cost minimization (Case-II).

Algorithm	Operating cost (€ct)						CPU time (s)
	Min	Max	Mean	Median	Variance	Std. dev	
SaCryStAl	53.92	349.74	56.79	109.25	17.8127	4.1805	551.684
CryStAl	54.75	371.28	57.46	112.37	18.1084	4.2358	573.841
HBA^115^	55.58	435.65	60.39	55.58	18.3	4.2779	614.053
DAOA^115^	56.676	535.89	134.53	139.22	18.681	4.3222	784.503
ARO^115^	55.724	630.61	67.508	56.44	18.417	4.2914	840.684
TDO^115^	55.582	939.29	63.146	56.291	18.301	4.278	1144.693
CHIO^115^	60.165	933.87	97.845	67.705	19.928	4.4641	1250.64
MRFO^115^	57.764	434.75	72.105	63.149	19.567	4.4234	1575.98
AO^115^	63.4926	722.509	278.832	337.697	22.131	4.7043	1584.589

**Table 9:** Statistical analysis of optimization results for emission minimization (Case-II).

Algorithm	Emission (kg)						CPU time (sec)
	Min	Max	Mean	Median	Variance	Std. dev	
SaCryStAl	135.186	439.048	138.905	154.49	0.5629	0.0237	597.062
CryStAl	138.205	447.204	140.752	157.282	0.5803	0.0243	599.604
HBA^115^	137.008	708.795	141.685	137.65	0.0006	0.0246	607.052
DAOA^115^	324.958	459.245	356.798	358.102	12.1629	3.4875	651.459
ARO^115^	145.945	600.313	157.656	146.073	0.6042	0.7773	790.374
TDO^115^	145.944	752.899	148.77	145.946	0.6042	0.7773	1069.83
AO^115^	146.876	744.895	207.053	207.112	0.5858	0.7654	1142.79
MRFO^115^	146.171	550.711	158.956	147.267	0.5985	0.7736	1176.13
CHIO^115^	159.634	658.708	211.261	187.347	0.9825	0.9912	1507.5

**Table 10:** Comparison of best compromise solution for Case-I and Case-II.

Algorithm		SaCryStAl	CryStAl	ALO^91^	FSAPSO^91^	Lexicographic optimization algorithm^110^
Case-I	Operating cost (€ct)	177.29	178.13	187.81	191.042	180.4
	Emission (kg)	469.92	471.89	473.12	721.076	529.3
Case-II	Operating cost (€ct)	112.02	113.46	NA	NA	NA
	Emission (kg)	196.15	198.63	NA	NA	NA

*NA* not available.

### Case-III: operation of microgrid integrated with PHEVs

**Figure 10:**
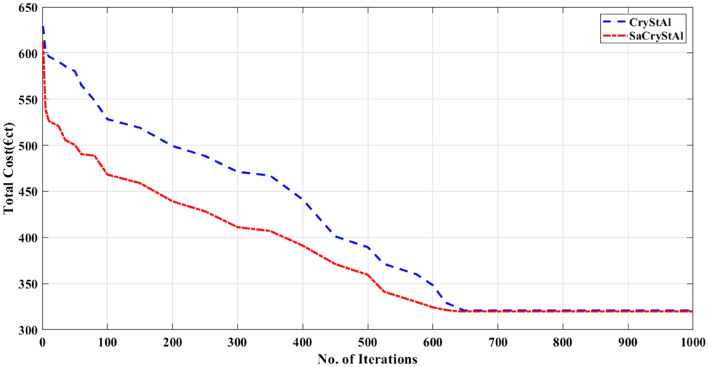
Convergence characteristic for the minimization of operating cost (uncoordinated charging mode).

**Figure 11:**
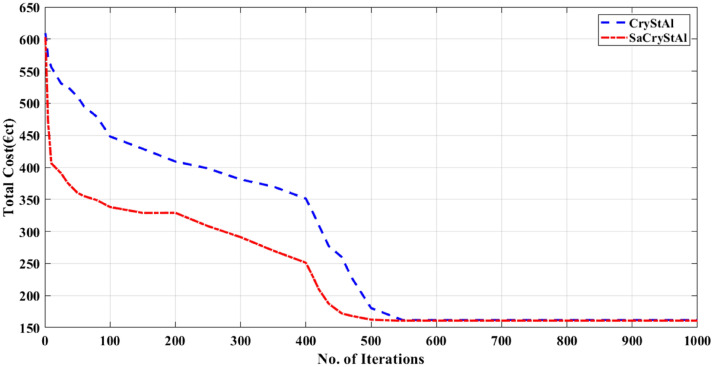
Convergence characteristic for the minimization of operating cost (coordinated charging mode).

**Figure 12:**
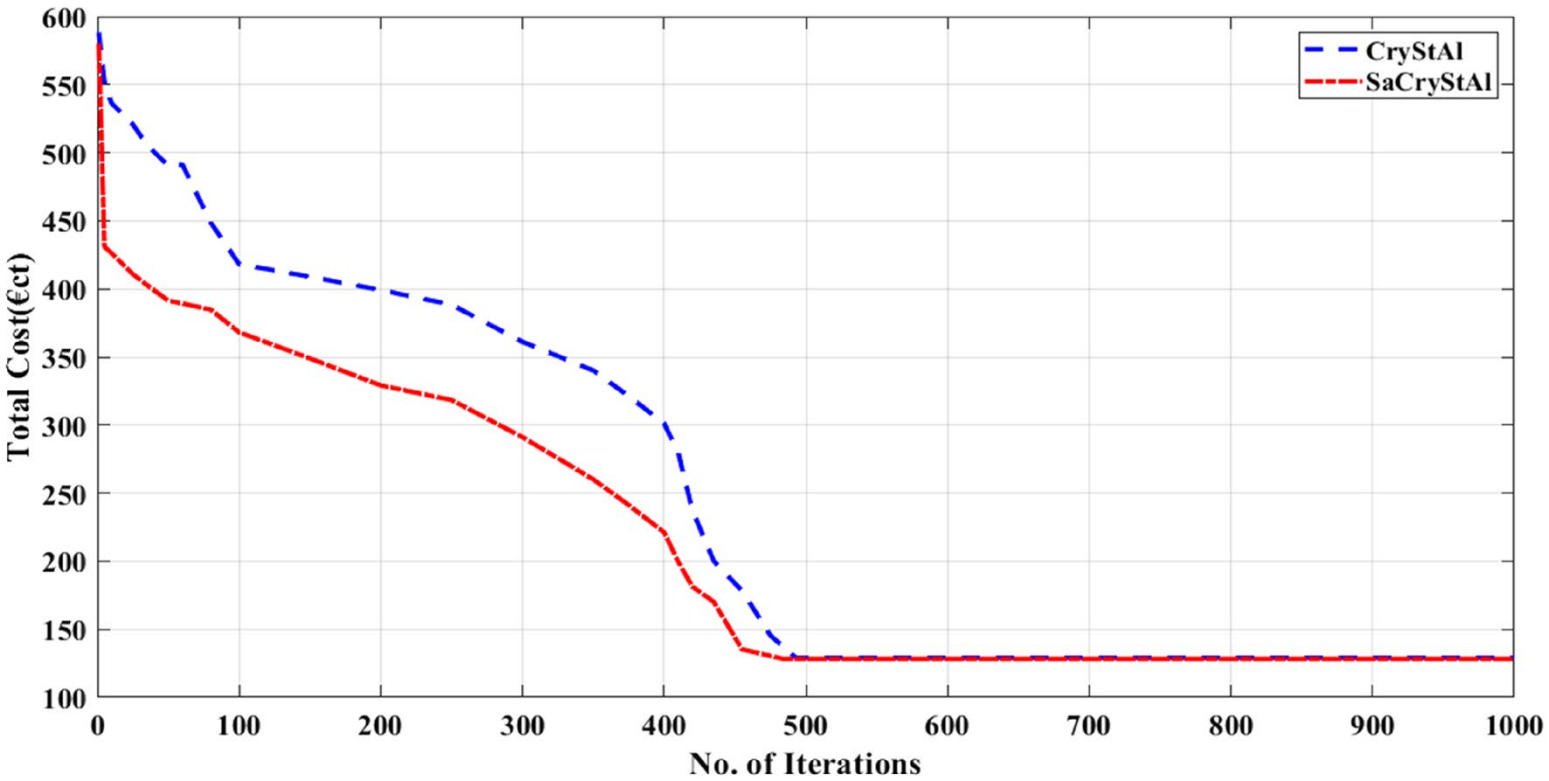
Convergence characteristic for the minimization of operating cost (smart charging mode).

In this analysis, the integration of PHEVs with the microgrid is explored. It is assumed that 30% of the 70 EVs are linked to the MG^77^. The objective in this scenario is to minimize operating costs. Here, the uncoordinated, coordinated, and intelligent charging modes of PHEVs are investigated. The optimal generation schedule of microgrid with the integration of PHEVs for the minimization of operating cost for uncoordinated, coordinated and smart charging modes are presented in Tables 11, 12 and 13 respectively. Table 14 presents the optimal value of the operation cost obtained using SaCryStAl and other optimization algorithms for the three different charging methods considered. It is evident from the optimization results, the proposed SaCryStAl performed better than CryStAl algorithm. In the uncoordinated, coordinated, and smart charging modes, the SaCryStAl algorithm attained optimal fitness values of 319.9301 €ct, 160.9827 €ct, and 128.2815 €ct, respectively. Figures 10, 11 and 12 present the convergence characteristics for the minimization of operating cost for uncoordinated, coordinated and smart charging modes respectively. The convergence behavior curve regarding operating cost minimization demonstrates that the proposed SaCryStAl algorithm exhibits smoother and more rapid convergence compared to the CryStAl algorithm across all three charging modes investigated. Moreover, Figs. 10, 11, and 12 illustrate that the SaCryStAl algorithm delivers swift and resilient performance, effectively mitigating optimization challenges encountered in diverse power systems. In order to supply the PHEVs from the MG, there is a limitation in the utility generating to acquire the full power capabilities. When using coordinated and smart charging modes, there is less integration between MT and the grid. As a result, both modes’ running costs are better than the uncoordinated charging mode’s. According to Tables 11, 12 and 13, the FC serves as the primary energy source during the day, with the grid being used at night and in the morning. MT generation is roughly constrained. The RESs and batteries assist in meeting the demand at midday, and any extra electricity is then sold to the grid. In this case, the recommended energy management technique outperformed the other optimizers taken into consideration to produce the best operating costs for MG with PHEVs.

**Table 11:** Optimal generation schedule for minimization of operating cost for Case-III (uncoordinated charging).

Hour	$P_{d}$(kW)	$P_{pv}$(kW)	$P_{wt}$(kW)	$P_{mt}$(kW)	$P_{FC}$(kW)	$P_{Batt}$(kW)	$P_{grid}$(kW)
1	52	0	2.39	29.99	22.5	− 30	59.99
2	50	0	2.42	29.99	5	− 18.91	60
3	50	0	2.51	29.75	21.52	− 27.75	57.25
4	51	0	2.48	29.62	28.3	− 27.75	50.16
5	56	0	2.44	29.64	21.88	− 20	54.75
6	63	0	1.22	29.99	30.99	− 31.99	59.99
7	70	0	2.43	29.99	31.65	− 19.72	59.99
8	75	0	2.37	29.99	30.55	− 32.97	59.99
9	76	4.29	4.99	29.91	34.99	58.91	− 11.92
10	80	7.51	10.55	29.82	37.75	59.34	− 19.93
11	78	10.31	19.25	29.82	36.51	59.34	− 19.99
12	74	19.25	22.55	29.74	46.73	44.21	− 23.29
13	72	22.65	27.72	29.74	34.2	47.23	− 17.56
14	72	21.99	22.61	28.63	35.25	47.23	− 21.78
15	76	13.25	10.12	28.59	37.61	59.91	− 19.97
16	80	4.99	7.43	28.67	36.25	59.32	− 18.02
17	85	1.43	2.55	29.89	32.55	60	− 7.99
18	88	0	2.43	29.75	32.46	60	90.02
19	90	0	2.35	29.99	32.41	60	90.02
20	87	0	2.53	29.99	32.52	60	90.02
21	78	0	2.31	29.99	32.48	60	81.75
22	71	0	2.36	29.99	32.53	60	77.62
23	52	0	2.12	29.99	32.47	33.28	70.19
24	50	0	1.55	29.99	32.63	0	59.99

**Table 12:** Optimal generation schedule for minimization of operating cost for Case-III (coordinated charging).

Hour	$P_{d}$(kW)	$P_{pv}$(kW)	$P_{wt}$(kW)	$P_{mt}$(kW)	$P_{FC}$(kW)	$P_{Batt}$(kW)	$P_{grid}$(kW)
1	52	0	2.41	29.98	22.4	− 29.99	60
2	50	0	2.41	29.98	21.78	− 29.99	59.02
3	50	0	2.41	29.79	22.75	− 27.86	57.29
4	51	0	2.41	29.65	28.75	− 22.79	50.11
5	56	0	2.41	29.67	9.88	− 9.16	57.18
6	63	0	1.24	29.98	31.11	− 29.97	60
7	70	0	2.43	29.98	32.08	− 21.09	60
8	75	0	2.38	29.98	30.78	− 17.93	60
9	76	4.29	5.11	29.89	37.41	58.94	− 18.92
10	80	7.51	10.57	29.81	38.72	57.98	− 18.92
11	78	10.31	19.33	29.79	41.12	51.92	− 20
12	74	19.25	22.47	26.72	56.72	56.94	− 25.29
13	72	22.65	27.72	24.74	32.11	40.03	− 22.98
14	72	21.99	22.63	18.23	39.27	57.93	− 17.99
15	76	13.25	10.14	23.39	36.78	59.96	− 24.39
16	80	4.99	7.39	28.67	35.75	59.48	− 17.79
17	85	1.43	2.53	29.89	32.49	59.99	− 4.99
18	88	0	2.41	29.75	31.24	59.99	− 3.28
19	90	0	2.34	29.94	30.89	2.13	60
20	87	0	2.57	29.94	30.02	59.99	− 4.82
21	78	0	2.32	29.94	30.91	59.99	80.93
22	71	0	2.34	29.94	30.34	59.99	77.18
23	52	0	2.13	29.94	30.31	25.78	70.21
24	50	0	1.53	29.98	30.12	0	60

**Table 13:** Optimal generation schedule for minimization of operating cost for Case-III (smart charging).

Hour	$P_{d}$(kW)	$P_{pv}$(kW)	$P_{wt}$(kW)	$P_{mt}$(kW)	$P_{FC}$(kW)	$P_{Batt}$(kW)	$P_{grid}$(kW)
1	52	0	2.44	30	22.78	− 30	60
2	50	0	2.44	30	20	− 30	60
3	50	0	2.44	28.39	5.12	− 7.98	58.13
4	51	0	2.44	30	32.12	− 5.48	60
5	56	0	1.29	30	32.12	− 1.28	60
6	63	0	2.43	30	31.78	− 29.99	60
7	70	0	2.09	30	31.29	− 21.18	60
8	75	0	2.43	30	33.13	− 17.89	60
9	76	5.15	2.39	29.12	32.78	59.02	− 19.18
10	80	7.63	6.99	28.29	37.01	56.29	− 17.28
11	78	10.09	18.98	23.91	45.12	51.18	− 21.89
12	74	12.89	21.99	18.12	49.14	47.12	− 24.71
13	72	24.92	28.19	27.97	51.29	49.12	− 27.89
14	72	20.18	22.87	30	44.89	49.89	− 30
15	76	9.12	10.09	28.92	38.19	57.92	− 19.94
16	80	5.23	7.59	30	32.01	59.78	− 9.83
17	85	1.46	2.71	30	31.29	60	− 3.75
18	88	0	2.48	30	30.98	60	− 2.16
19	90	0	2.33	30	31.89	− 1.89	60
20	87	0	2.48	30	32.98	60	− 2.56
21	78	0	2.43	30	31.87	60	− 9.99
22	71	0	2.43	30	31.87	60	− 20
23	52	0	2.09	30	30.99	− 27.13	60
24	50	0	1.49	30	27.99	− 30	60

**Table 14:** Analysis of simulation results for minimizing operating costs across three charging modes.

Algorithm	Uncoordinated charging (€ct)	Coordinated charging (€ct)	Smart charging (€ct)
SaCryStAl	319.9301	160.9827	128.2815
CryStAl	320.8627	161.9064	129.0953
GSA-PS^78^	675.4259	390.4521	337.2845
BES^78^	321.7595	162.7251	129.8758
RUN^78^	322.0152	164.2675	131.5451
MGO^78^	322.2636	162.7319	132.6798
CBOA^78^	407.3604	242.3315	197.7891
BWO^78^	327.7516	175.3402	148.7032
DO^78^	328.1547	178.6087	142.4507

## Conclusion and future research directions

This research implemented a new energy management technique for MGs with installed RESs and PHEVs that incorporates the SaCryStAl algorithm. The suggested method is accountable for distributing energy among many units. In the grid-connected MG, fuel cells, storage batteries, plug-in hybrid electric vehicles (PHEVs), nonrenewable sources (MT), and renewable generators (PV and WT) are all taken into consideration. The objectives taken into account in this effort include lowering the MG operating cost and reducing pollutant emission. In order to compare the performance of the proposed algorithm to currently used evolutionary optimization approaches, the study took into account three different scenarios. The research investigated three different scenarios to assess the effectiveness of the proposed algorithm compared to conventional CryStAl and other optimization techniques. The authors conducted simulations for each scenario and compared the results. In the first scenario, the SaCryStAl algorithm, designed for single-objective optimization, successfully achieved optimal solutions for cost and emissions, recording 124.15 €ct and 419.14 kg, respectively, within acceptable time frames of 97.19 and 76.41 s respectively. Optimization result surpassed those of other existing optimization algorithms. In the second scenario, the SaCryStAl algorithm once again provided superior results, delivering optimal cost and emission values of 53.92 €ct and 135.186 kg, respectively, within acceptable computational times of 551.684 and 597.062 s respectively. In the third scenario, the SaCryStAl algorithm maintained its success by achieving optimal operation costs of 319.9301 €ct, 160.9827 €ct, and 128.2815 €ct for the uncoordinated, coordinated, and smart charging modes of PHEVs, respectively. Moreover, the SaCryStAl algorithm demonstrated strong performance in optimizing both cost and emissions within a multi-objective framework. In the first scenario, it achieved optimal operational cost and emissions of 177.29 €ct and 469.92 kg respectively. In the second scenario, the algorithm produced even better results with optimal operational cost and emissions of 112.02 €ct and 196.15 kg respectively. The study’s findings suggest that widespread use of PHEVs and RES will have a significant impact on grid functioning in terms of emission goals. The SaCryStAl algorithm demonstrates remarkable stability, convergence, and performance, as evidenced by the numerical results. Notably, it yields a diverse collection of Pareto-optimal solutions that are evenly distributed. This abundance of options empowers system operators to select the most suitable power dispatch strategy to meet their economic and environmental objectives effectively. Furthermore, our proposed method outperforms other optimization algorithms in terms of both economic and environmental outcomes. Remarkably, despite its superior performance, the computational time of our approach remains practically identical to that of the conventional CryStAl. Moreover, our current research extends beyond mere optimization by incorporating market pricing, load, photovoltaic (PV), and wind turbine (WT) uncertainties. This holistic approach ensures the optimal scheduling of microgrid operations, considering real-world uncertainties and enhancing the robustness of our findings.

In the future, a stochastic model that takes into account hydrothermal units as well as renewable energy sources could be offered. The proposed model’s influence on pollutant emissions can be thoroughly examined, and market prices and tariff structures can also be taken into account. In considering future research directions, several promising avenues emerge from this study’s findings. Firstly, further investigation into the integration of emerging technologies, such as advanced energy storage systems and demand response mechanisms, could enhance the efficiency and resilience of microgrid operations. Additionally, exploring the applicability of the proposed SaCryStAl algorithm in larger-scale energy systems and diverse geographical contexts would be beneficial. Furthermore, incorporating real-time data analytics and machine learning techniques could augment the algorithm’s decision-making capabilities, enabling more adaptive and proactive energy management strategies. Lastly, exploring the socio-economic implications of microgrid integration and assessing the potential barriers to adoption could provide valuable insights for policymakers and industry stakeholders. By addressing these research avenues, future studies can contribute to advancing the state-of-the-art in microgrid optimization and facilitating the transition towards sustainable and resilient energy systems.

## Data Availability

The datasets used and/or analysed during the current study available from the corresponding author on reasonable request.

## Abbreviations

AER: All-electric range
ASAPSO: Adaptive simulated annealing particle swarm optimization algorithm
BES: Bald eagle search
BESS: Battery energy storage system
BS: Battery storage
BWO: Beluga whale optimization
CBOA: Chef-based optimization algorithm
CHP: Combined heat power
CO_2_: Carbon dioxide
DERs: Distributed energy resources
DGs: Distributed generators
DO: Dandelion optimizer
DR: Demand response
DRP: Demand response program
EMS: Energy management strategy
EV: Electric vehicle
FC: Fuel cell
FSAPSO: Fuzzy self-adaptive PSO
GA: Genetic algorithm
GSA-PS: Gravitational search and pattern search
GWO: Grey wolf optimizer
HRES: Hybrid renewable energy system
HS: Heat storage
KH: Krill herd algorithm
LV: Low voltage
MDP: Markov decision process
MG: Microgrid
MGCC: Microgrid central controller
MGO: Mountain gazelle optimizer
MGT: Micro gas turbine
MPC: Model predictive control
MT: Microturbine
MV: Medium voltage
NA: Not available
Nox: Nitrogen dioxide
OGGWO: Oppositional gradient-based grey wolf optimizer
PAFC: Phosphoric acid fuel cell
PDF: Probability density function
PEV: Plug-in electric vehicles
PHEV: Plug-in hybrid electric vehicle
PLR: Peak load reduction
PSO: Particle swarm optimization
PV: Photovoltaic
RES: Renewable energy sources
RUN: Runge Kutta optimization
SaCryStAl: Self-adaptive crystal structure algorithm
SO_2_: Sulfur dioxide
SOC: State of charge
WOA: Whale optimization algorithm
WT: Wind turbine

## List of symbols

$$N$$: Population size
N: Maximum no. of iterations
$h$: Total number of hours
$N_{DG}$: Total number of distributing generation units
$N_{ST}$: Total number of storage units
$N_{KL}$: Total number of load levels
$U_{i} \left( h \right)$: Status of unit $i$ at hour ${\text{h}}$
$P_{DGi} \left( h \right)$: Active power output of $i{\text{th}}$ distributed generating unit at hour ${\text{h}}$
$P_{STj} \left( h \right)$: Active power output of $j{\text{th}}$ storage unit at hour ${\text{h}}$
$P_{GR} \left( h \right)$: Active power exchanged with the grid through buying or selling at hour ${\text{hr}}$
$B_{DGi} \left( h \right)$: The bid of the $i{\text{th}}$ distributed generation (DG) source during hour ${\text{h}}$
$B_{STj} \left( h \right)$: The bid of the $j{\text{th}}$ storage option during hour ${\text{h}}$
$B_{GR} \left( h \right)$: The bid of grid during hour ${\text{h}}$
$S_{DGi}$: Costs associated with starting up or shutting down the $i{\text{th}}$ distributed generation (DG) unit
$S_{STj}$: Costs associated with starting up or shutting down the $j{\text{th}}$ storage unit
$E_{DGi} \left( h \right)$: Emission in ${\text{kg}}/{\text{MWh}}$ for $i{\text{th}}$ distributed generation (DG) unit during hour ${\text{h}}$
$E_{STj} \left( h \right)$: Emission in ${\text{kg}}/{\text{MWh}}$ for $j{\text{th}}$ storage unit during hour ${\text{h}}$
$E_{GRD} \left( h \right)$: Emission in ${\text{kg}}/{\text{MWh}}$ for grid during hour ${\text{h}}$
${\text{CO}}_{{2_{{DG_{i} }} }} \left( h \right)$: Carbon dioxide emissions from the $i{\text{th}}$ distributed generation (DG) unit during hour ${\text{h}}$
${\text{SO}}_{{2_{{DG_{i} }} }} \left( h \right)$: Sulphur dioxide emissions from the $i{\text{th}}$ distributed generation (DG) unit during hour ${\text{h}}$
${\text{NO}}_{{X_{{DG_{i} }} }} \left( h \right)$: Nitrogen oxide emissions from the $i{\text{th}}$ distributed generation (DG) unit during hour ${\text{h}}$
${\text{CO}}_{{2_{{ST_{j} }} }} \left( h \right)$: Carbon dioxide emissions from the $j{\text{th}}$ storage unit during hour ${\text{h}}$
${\text{SO}}_{{2_{{ST_{j} }} }} \left( h \right)$: Sulphur dioxide emissions from the $j{\text{th}}$ storage unit during hour ${\text{h}}$
${\text{NO}}_{{X_{{ST_{j} }} }} \left( h \right)$: Nitrogen oxide emissions from the $j{\text{th}}$ storage unit during hour ${\text{h}}$
${\text{CO}}_{{2_{GRD} }} \left( h \right)$: Carbon dioxide emissions from the grid during hour ${\text{h}}$
$SO_{{2_{GRD} }} \left( h \right)$: Sulphur dioxide emissions from the grid during hour ${\text{h}}$
${\text{NO}}_{{X_{GRD} }} \left( h \right)$: Nitrogen oxide emissions from the grid during hour ${\text{h}}$
$P_{LLk}$: The quantity of the $k{\text{th}}$ load level
$N_{KL}$: Total number of load levels
$P_{DG,min} \left( h \right)$: Minimum active power generation of $i{\text{th}}$ DG during hour $h$
$P_{ST,min} \left( h \right)$: Minimum active power generation of $j{\text{th}}$ storage unit during hour ${\text{h}}$
$P_{GR,min} \left( h \right)$: Minimum active power generation of grid during hour ${\text{h}}$
$P_{DG,max} \left( h \right)$: Maximum active power generation of $i{\text{th}}$ DG during hour ${\text{h}}$
$P_{ST,max} \left( h \right)$: Maximum active power generation of $j{\text{th}}$ storage unit during hour ${\text{h}}$
$P_{GR,max} \left( h \right)$: Maximum active power generation of grid during hour ${\text{h}}$
$E_{b} \left( h \right)$: Energy stored in the battery at time ${\text{h}}$
$P_{ch} /P_{disch}$: Rate of charge/discharge allowed over a specific timeframe.
$\xi_{ch} /\xi_{disch}$: Battery efficiency in the process of charging and discharging.
$E_{b}^{min} /E_{b}^{max}$: Limits for the storage capacity of the battery, both minimum and maximum.
$P_{ch,max} /P_{disch,max}$: The highest permissible charge/discharge rate within each time segment, represented as the inertia weight parameter
$R_{up}^{i}$: Ramp-up of the $i{\text{th}}$ DG output power
$R_{down}^{i}$: Ramp-down of the $i{\text{th}}$ DG output power
$$\Delta h$$: Time step
$$w$$: Weighting factor
$\rho$: Price penalty factor
